# Merging Heterogeneous Graphitic Carbon Nitride Photocatalysis with Cobaloxime Catalysis in Uphill Dehydrogenative Synthesis of Anilines

**DOI:** 10.1002/cssc.202402439

**Published:** 2025-04-09

**Authors:** Sonia Zoltowska, Stefano Mazzanti, Sara Stolfi, Jingsan Xu, Matej Huš, Ana Oberlintner, Matic Pavlin, Paolo Ghigna, Blaž Likozar, Piero Torelli, Luca Braglia, Davide Ravelli, Maurizio Fagnoni, Iker Agirrezabal‐Telleria, Markus Antonietti, Paolo Giusto, Oleksandr Savateev

**Affiliations:** ^1^ Department of Colloid Chemistry Max Planck Institute of Colloids and Interfaces Am Mühlenberg 1 D‐14476 Potsdam Germany; ^2^ Department of Chemistry University of Pavia viale Taramelli 12 27100 Pavia Italy; ^3^ TASC Laboratory CNR–IOM Istituto Officina dei Materiali 34149 Trieste Italy; ^4^ School of Chemistry and Physics Queensland University of Technology Brisbane Australia; ^5^ Department of Catalysis and Chemical Reaction Engineering National Institute of Chemistry Hajdrihova 19 1000 Ljubljana Slovenia; ^6^ Association for Technical Culture of Slovenia (ZOTKS) Zaloška 65 1000 Ljubljana Slovenia; ^7^ Research Institute Institute for the Protection of Cultural Heritage of Slovenia (ZVKDS) Poljanska 40 1000 Ljubljana Slovenia; ^8^ Department of Chemical and Environmental Engineering of the Bilbao Engineering School University of Basque Country (UPV/EHU) Plaza Torres Quevedo 1 Bilbao 48013 Spain; ^9^ Department of Chemistry The Chinese University of Hong Kong Sha Tin, New Territories Hong Kong China

**Keywords:** carbon nitride, hydrogen, organic synthesis, photocatalysis, X‐ray absorption spectroscopy

## Abstract

Synthesis of substituted anilines upon nucleophilic addition of secondary amines to cyclohexanone derivatives followed by aromatization of the enamine by employing a combination of Ir‐polypyridine complex as a photoredox catalyst and cobaloxime as H_2_‐evolution catalyst is developed recently by Leonori et al. In this work, the homogeneous photoredox catalyst is replaced by a heterogeneous and metal‐free mesoporous graphitic carbon nitride (mpg‐CN). Substituted aromatic amine and H_2_ are formed simultaneously. Combination of X‐ray spectroscopies reveals charge transfer from cobaloxime to mpg‐CN in the dark. Illumination of the catalytic system with visible light induces electron transfer from mpg‐CN to cobaloxime and formation of persistent Co(II) species. The results of density functional theory modeling suggest that the studied reaction is strongly endothermic and endergonic. Thus, energy of photons is stored in the reaction products—H_2_ and the aromatic amine.

## Graphical abstract

Heterogeneous photocatalyst, mesoporous graphitic carbon nitride (mpg‐CN) and H_2_‐evolution catalyst, cobaloxime, enable endergonic and endothermic reaction—synthesis of substituted anilines along with H_2_ evolution.

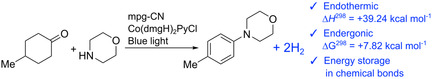

## 1 Introduction

**Figure 1:**
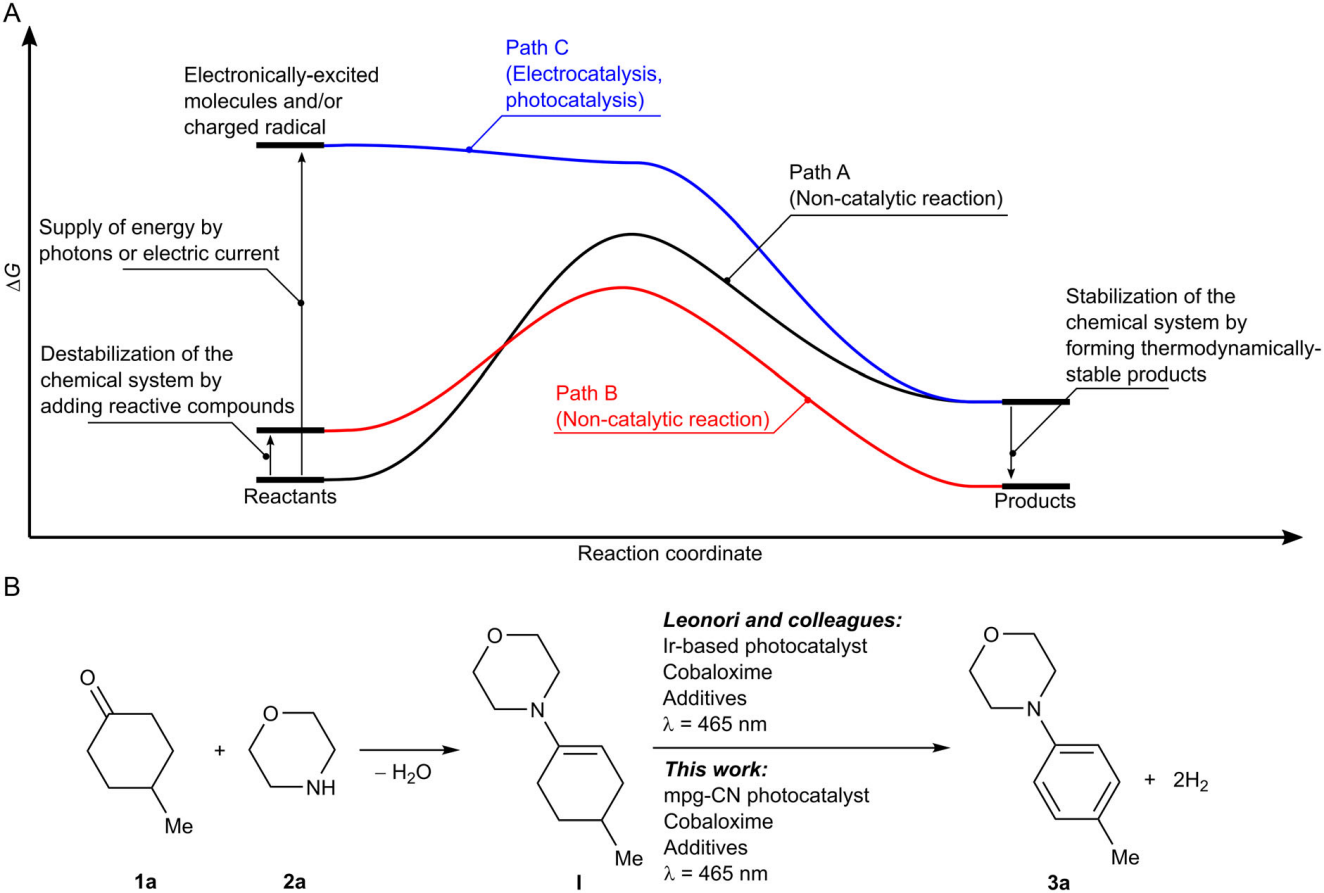
A) Path A denotes schematically a reaction profile of a certain endergonic reaction and synthesis of a target compound. Path B denotes synthesis of the target compound when it is coupled with another exergonic reaction. Path C denotes photochemical, electrochemical approaches or plasma catalysis that involve formation of reactive intermediates—radical ions and electronically excited states. B) Synthesis of anilines from aliphatic ketones and amines that is accompanied by H_2_ evolution reported by Leonori and colleagues.

The second law of thermodynamics allows predicting if a certain chemical reaction is spontaneous or non‐spontaneous in the given conditions. In practice, it requires computing and/or measuring change of enthalpy (Δ*H*), entropy (Δ*S*), and computation of Gibbs free energy (Δ*G*) of a chemical system that undergoes a chemical reaction. Many chemical reactions are characterized by positive change in Δ*G*, making the reaction thermodynamically non‐spontaneous (**Figure** **1**, path A). There are several approaches that enable such intrinsically endergonic reactions. For instance, a commonly exploited approach in chemistry laboratories involves coupling of a desired intrinsically endergonic reaction (Δ*G*_*i*_ > 0) with another highly exergonic one (Δ*G*_*j*_ < 0) so that Δ*G*_*j*_ < ‐Δ*G*_*i*_ –overall Gibbs free energy change of the sum of these two reactions is negative (Figure 1, path B). In practice, compounds with weakly negative or even positive enthalpy of formation are added to the reaction mixture. The energy that is released upon their conversion into thermodynamically more stable compounds is employed to drive the desired intrinsically uphill reaction. However, atom efficiency of this approach in general is low. For example, when metals are used as reductants, they are converted into metal oxides and salts, a chemical waste. More innovative, but at the same time more challenging approach, is to lift the Gibbs free energy of the reactants, to afford Δ*G* < 0 (Figure 1, path C),^[^ ^1^, ^2^ ^]^ which is realized in electrolysis (or electrocatalysis), photochemistry (or photocatalysis), and plasma catalysis.^[^ ^3^ ^]^ Typically, charged radical species generated upon oxidation, reduction or ionization of substrates and electronically excited state of compounds obtained upon photon absorption are highly reactive. Therefore, in general, they undergo transformations into more stable species spontaneously. Despite overcoming the activation energy barrier, Δ*G* remains positive, meaning the reaction is thermodynamically non‐spontaneous. Therefore, to prevent the reaction from reversing, an appropriate strategy must be employed. For instance, in water splitting, the reverse reaction (H_2_ + O_2_ → H_2_O) should be negligible because the recombination barrier is also suppressed.^[^ ^4^ ^]^ However, if H_2_ is generated as a product, its removal prevents the reverse reaction, enabling energy storage in the form of chemical bonds. It is important to note that not all reactions can be efficiently driven by photocatalysis, as many will naturally revert unless an energy storage mechanism is in place.^[^ ^5^ ^]^

One of the most studied intrinsically endergonic reactions is water dissociation into H_2_ and O_2_. This reaction does not occur spontaneously under the standard conditions, the Δ*G* ^298^ of the reaction 2H_2_O → 2H_2_ + O_2_ is +113 kcal mol^−1^. However, the reaction proceeds when energy is supplied by photons or electromotive force. When this energy in the form of electricity for electrolysis or light for photocatalysis is derived from renewable sources, the generated H_2_ is considered *green*. While the by‐product of water splitting, O_2_, is less valuable, electrocatalytic or photocatalytic water splitting can afford oxygen of high purity to complement the existing air separation technology. An alternative approach considers generation of H_2_ from hydrogen‐rich organic compounds, coupled with their conversion into value‐added products. The examples of products synthesized via this approach are represented by chemical commodities, such as acetaldehyde^[^ ^6^, ^7^, ^8^, ^9^, ^10^ ^]^ or 2,3‐butandiol^[^ ^8^, ^11^, ^12^, ^13^ ^]^ that are obtained upon dehydrogenation of ethanol, ethylene glycol—from methanol,^[^ ^14^, ^15^, ^16^, ^17^ ^]^ and other organic compounds.^[^ ^18^, ^19^, ^20^ ^]^ It is worth to mention that the demand for such fine organic compounds is significantly lower compared to the aforementioned industrial commodities, H_2_ and O_2_. Given that H_2_ and organic compounds are generated in stoichiometric ≈1:1 ratio, various oxidant‐free cross‐dehydrogenative coupling^[^ ^21^ ^]^ and cross‐coupling hydrogen evolution reactions^[^ ^22^ ^]^ cannot, in principle, fulfill all the demand of H_2_. Nevertheless, these strategies to construct more complex organic compounds are more atom efficient – i) prefunctionalization of the organic substrates is avoided, ii) the “by‐products” are H_2_, H_2_O, CO_2_, and/or other small molecules.^[^ ^17^, ^23^, ^24^, ^25^, ^26^, ^27^, ^28^, ^29^ ^]^ More promising is the generation of H_2_ from biomass, such as lignin, cellulose,^[^ ^30^ ^]^ or pollutants.^[^ ^31^ ^]^ However, the yield rate of H_2_ is significantly lower compared to model small organic sacrificial molecules—glucose, and other sugars.^[^ ^32^ ^]^ It is also of fundamental interest to expand the scope of dehydrogenation reactions and identify which of them are intrinsically endergonic, but are enabled by means of photocatalysis. In this case the energy of photons is stored in the organic products, while the reaction is regarded as artificial photosynthesis.^[^ ^33^ ^]^

Recently, Leonori et al. developed an elegant approach to synthesize substituted anilines from aliphatic ketones and amines employing a combination of Ir‐polypyridine photoredox catalyst and Co(dmgH)_2_(Me_2_NPy)Cl (Figure 1B).^[^ ^34^ ^]^ The method shows several advantages—i) there are many precursors available commercially, and ii) in addition to substituted anilines two H_2_ and one H_2_O equivalents are generated as the by‐products. On the contrary, i) it relies on Ir‐based photoredox catalyst—this transition metal is rare and expensive, and ii) endo‐/exoergicity of the reaction was not assessed. These aspects prompted us to gain the missing pieces of information and to develop a complimentary method that does not require Ir complexes as photocatalyst.

Herein, we present a method of anilines synthesis from aliphatic ketones and amines that is enabled by mesoporous graphitic carbon nitride (mpg‐CN) as the heterogeneous photocatalyst coupled with commercial Co(dmgH)_2_PyCl as H_2_‐evolution catalyst.

## 2 Results and Discussion

First, the thermodynamics of the reactions was calculated to ascertain their feasibility and spontaneity. The condensation of **1a** and **2a** into **I** is thermoneutral for practical purposes (Δ*H* ^298^ = +1.56 kcal mol^−1^) and slightly endergonic (Δ*G* ^298^ = +2.88 kcal mol^−1^) in standard conditions (Figure 1B). The equilibrium constant can be shifted by changing the temperature and the equilibrium composition can be influenced by changing the fraction of reactants and products, including water. Thus, the step is not a thermodynamic bottleneck. A subsequent conversion of **I** into **3a**, where two molecules of H_2_ are also formed, is strongly endothermic (Δ*H* ^298^ = +39.24 kcal mol^−1^). The formation of two hydrogen molecules increases the entropy of the system, offsetting some increase in the Gibbs free energy (Δ*G* ^298^ = +7.82 kcal mol^−1^). Thus, a fraction of photon energy is stored in the products.

We studied synthesis of **3a** from 4‐methylcyclohexanone **1a** as a saturated aryl surrogate and morpholine **2a**. Their interaction with the formation of **I** occurs spontaneously, in agreement with the results of thermodynamic calculations. Following the procedure proposed by Leonori and colleagues^[^ ^34^ ^]^ we tested a catalytic system based on mpg‐CN and Co(dmgH)_2_PyCl complex (Figure 1B). In our reaction system, mpg‐CN serves as the photocatalyst, whose electronically excited state is quenched reductively by **I**, while Co(dmgH)_2_PyCl functions as the dehydrogenation catalyst. The results of the reaction conditions optimization are summarized in **Table** **1**.

**Table 1:** Screening of the reaction conditions of aniline synthesis.

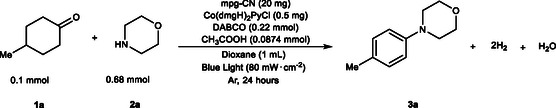		
Entry	Deviations from standard conditions	Yield [%]
1	Noneb)	49a)
2	Na‐PHI	9
3	Without mpg‐CN	n.d.
4	Without Co(dmgH)_2_PyCl	n.d.
5	In the dark	n.d.
6	mpg‐CN; Purple LEDc)	16
7	mpg‐CN; UV LEDd)	27
8	mpg‐CN; Co(dmgH)_2_PyCl (0.0024 mmol)	48
9	mpg‐CN; Co(dmgH)_2_PyCl (0.0048 mmol)	50
10	mpg‐CN (30 mg)	44
11	mpg‐CN (10 mg)	46
12	mpg‐CN (5 mg)	32
13	mpg‐CN; air	28
14	mpg‐CN; without DABCO	10
15	mpg‐CN; without AcOH	30

a) GC yield;

b) Reaction conditions: ketone (0.1 mmol, 0.125 mL), morpholine (0.68 mmol, 0.59 mL), mpg‐CN 20 mg, Co(dmgH)_2_PyCl (0.0012 mmol, 0.5 mg), DABCO (0.22 mmol, 25 mg), glacial AcOH (0.0874 mmol, 0.005 mL), dioxane (1 mL), blue LEDs (*λ*_max_ = 460 nm, 80 mW cm^−2^), 25 °C, 24 h, n.d. – not detected;

c) *λ*_max_ = 400 nm, 60 mW cm^−2^;

d) *λ*_max_ = 365 nm, 60 mW cm^−2^.

The yield of aniline **3a** synthesized under the optimal reaction conditions reaches 49%. The screening of the reaction conditions indicates the crucial role of DABCO as a proton shuttle, which might be involved in the photocatalytic cycle as well as cobaloxime turnover step. Furthermore, the presence of acid and the absence of oxygen are necessary to obtain a satisfactory product yield. No product is obtained without the addition of mpg‐CN or Co(dmgH)_2_PyCl, highlighting their importance for the synthesis of **3a**. Similarly, no product is observed when the reaction is performed in the dark, demonstrating the photochemical nature of the process. Notably, increasing the amount of Co(dmgH)_2_PyCl does not significantly enhance the yield of **3a**, highlighting that mpg‐CN is not merely a photosensitizer but plays an active role in modulating the reaction pathway. This underscores the necessity of the cooperative interaction between mpg‐CN and Co(dmgH)_2_PyCl to drive the catalytic cycle effectively.

Stability of a catalytic system is crucial to evaluate its lifespan, resistance to deactivation, and overall durability, which are essential for maintaining consistent performance. Therefore, the reusability tests have been performed with and without the refilling of Co(dmgH)_2_PyCl. Details can be found in Supplementary note 1. The results indicate that mpg‐CN may be reused, but deactivation of Co(dmgH)_2_PyCl occurs. Therefore, addition of a fresh portion of Co(dmgH)_2_PyCl is required. Generation of H_2_ and aniline simultaneously was confirmed in the experiment conducted on 7 mmol scale of 4‐methylcyclohexanone resulting in formation of aniline with 35% yield (Figure S2, Supporting Information). A combination of mpg‐CN and Co(dmgH)_2_PyCl mediates the synthesis of anilines by coupling various aliphatic ketones with amines (Figure S5 and Table S1, Supporting Information). Small‐scale experiments demonstrated that the catalytic system is compatible with both saturated and unsaturated cyclic ketones. However, the observed isolated yields were lower than 30%. In a 5 mmol scale reaction with three different ketones, a measurable amount of hydrogen was produced, but the overall yields after 24 h remained lower than reported in Table 1, **entry 1**. These results suggest that interactions with mpg‐CN may play a role in yield reduction, as aromatic products could adsorb onto the catalyst surface, limiting its availability for further turnover.

It must be pointed out that, the replacement of Ir(dtbbpy)(ppy)_2_]PF_6_ with mpgCN as the photocatalyst and Co(dmgH)_2_(DMAP)Cl with Co(dmgH)_2_PyCl could influence the reactivity. While Leonori's system demonstrated reactivity ranging from 42 to 96% across various ketones,^[^ ^34^ ^]^ the current study achieved lower yields, with a maximum of 49% under the optimized conditions. The primary goal of this research is to assess the applicability of the mpgCN/Co(dmgH)_2_PyCl system in amination reactions, with a more detailed mechanistic investigation of this heterogeneous system centered on endo‐/exoergicity and energy storage in a chemical form, primarily due to generation of H_2_. The development of a more efficient catalytic system is beyond the scope of this study.

Therefore, the first step to understand the reaction mechanism involves elucidation of the roles of the mpg‐CN and the Co(dmgH)_2_PyCl. Consequently, a comprehensive characterization of the physicochemical properties of these materials was conducted. Detailed results of these characterizations are provided in Supplementary note 3.

**Figure 2:**
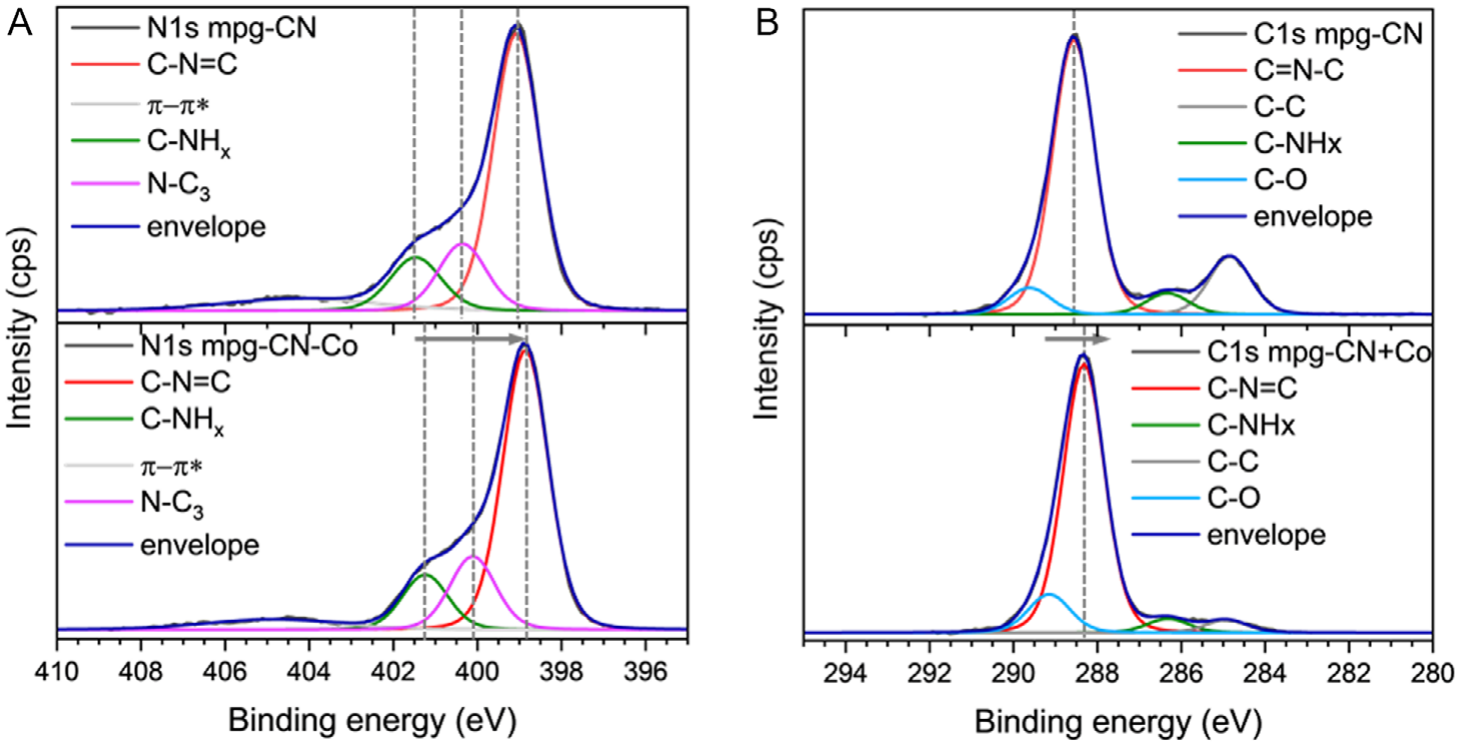
XPS analysis of mpg‐CN and Co(dmgH)_2_PyCl/mpg‐CN: A) N 1s, B) C 1s.

**Figure 3:**
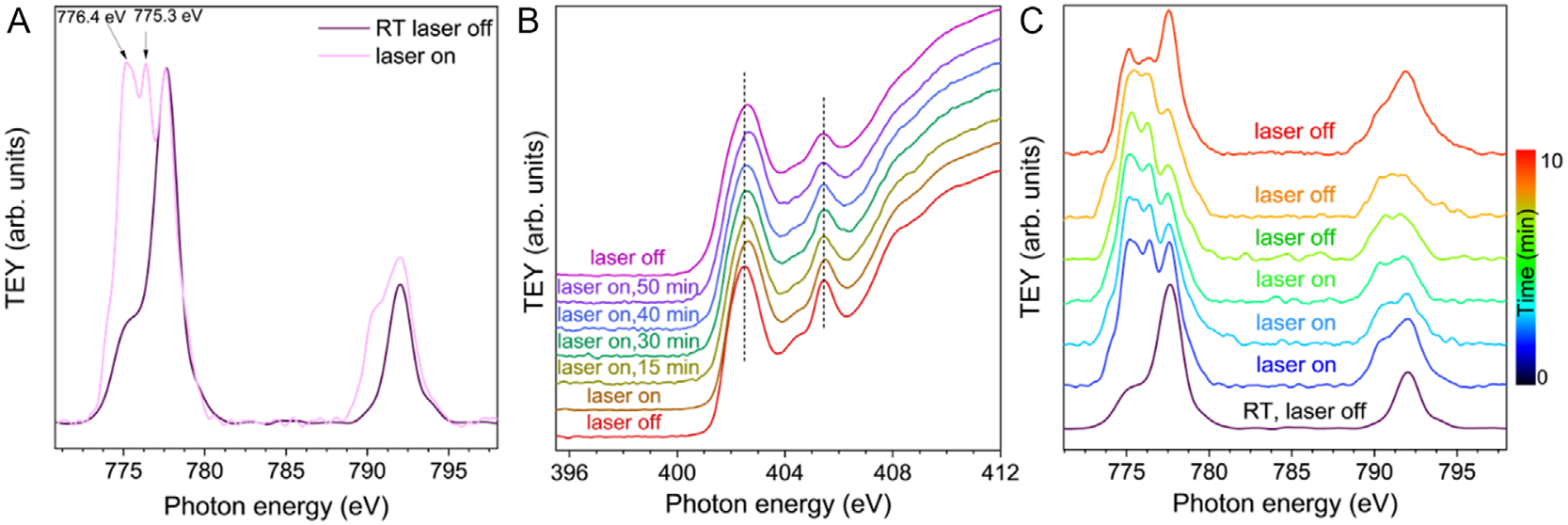
A) Co L_2,3_‐edge spectra of and Co(dmgH)_2_PyCl/mpg‐CN without (purple line) and with illumination (pink line), B) N K‐edge spectra without (red line), and C) Co L_2,3_‐edge spectra with illumination (different colors), and with the laser switched off after illumination (red line).

X‐ray absorption spectroscopy (XAS) and X‐ray photoelectron spectroscopy (XPS) were employed to get insights into the reaction mechanism and the electronic properties of the bare mpg‐CN and the composite Co(dmgH)_2_PyCl (2.4 wt%)/mpg‐CN, which was prepared by concentrating a mixture of mpg‐CN and Co(dmgH)_2_PyCl in organic solvent in vacuum. The mass fraction of cobaloxime in Co(dmgH)_2_PyCl/mpg‐CN is equal to that in entry 1 of Table 1. As shown by N 1*s* XPS spectrum, the introduction of Co(dmgH)_2_PyCl results in a shift of the peaks assigned to the C—N=C and N—C_3_ moieties in mpg‐CN by ≈0.3 eV and C—NH_*X*_ species by ≈0.2 eV to lower binding energies (**Figure** **2**). Such shift points to a charge redistribution from Co(dmgH)_2_PyCl to the mpg‐CN. A similar charge transfer direction from cobaloxime to mpg‐CN, along with a comparable peak shift magnitude (≈0.2–0.3 eV), is observed in the O 1*s* XPS spectrum (see Figure S10, Supporting Information). In contrast, in the C 1*s* spectrum, the peak shift is smaller (≈0.2 eV), likely due to the weaker influence of electron density redistribution on carbon atoms, which may result from weaker interactions, such as π–π interactions. Although XPS provides detailed information on the surface binding schemes and the electron density changes at the materials surface chemical features, X‐ray absorption near edge structure (XANES) provides complementary information especially for the study of the oxidation state changes at the Co L‐edge, before and after the reaction (**Figure** 3).

The XANES spectrum Co(dmgH)_2_PyCl/mpg‐CN in dark reveals that Co is in the oxidation state +3 (Figure 3A). Upon illumination of the sample with visible light (405 nm) we recorded a major change in the Co peak shape with the arising of two peaks located at 776.4 and 775.3 eV in the L_3_ edge spectrum pointing to a reduction of Co(III) to Co(II). At the N K‐edge, complementary changes are observed: the spectrum shifts toward higher energy values upon illumination, pointing to a charge transfer from the mpg‐CN to the cobalt. Thus, the edge energy position in the XANES spectrum is a measure of the ionization potential of the corresponding N core level (1*s* for the K‐edge). This is shielded by all the other (valence) electrons, and therefore a shift of the edge position toward higher energy indicates less shield and therefore, in chemical terms, an oxidation. The described changes in N K‐edge and Co L‐edge spectra are at least partially reversible: cessation of light irradiation leads to slow partial recovery of Co(III) and charge transfer back to mpg‐CN. However, it is worth mentioning that some changes, after prolonged irradiation, are not reversed (in the time of the experiment). For example, at the N K‐edge, there is a broadening of both the main peak at ≈402.5 eV and of the peak at ≈405.5 eV, that is not reversed.^[^ ^35^ ^]^ In summary, the XAS results are consistent with a scenario where the UV photons create electron–hole couples, where the hole is localized on mpg‐CN, while the electron is localized on the Co of the cobaloxime. Co(II) species are long‐lived and may be the resting state of cobaloxime. X‐ray absorption spectroscopy studies of Co‐polypyridyl complex and Co(dmgBF_2_)_2_ identified Co(I) species, which are formed transiently upon Co‐complex excitation in the presence of sacrificial electron donors—ascorbic acid or triethanolamine.^[^ ^36^, ^37^ ^]^ These conditions, however, are different from that employed in our current study—Co(dmgH)_2_PyCl/mpg‐CN was investigated in solid state in flowing He without adding any sacrificial agents.

**Figure 4:**
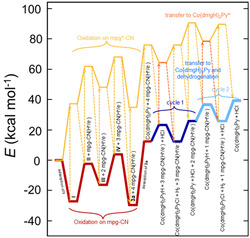
The energy profile for oxidation of **I** and dehydrogenation of mpg‐CN(H^+^/e^−^) by Co(dmgH)_2_PyCl to produce H_2_. Vertical lines represent excitation of a chemical system upon photon absorption.

Theoretical calculations were conducted to elucidate the reaction mechanism in more detail. First, **I** is adsorbed into pores on the (**ac**) surface of mpg‐CN with an adsorption interaction of 1.18 eV (27.1 kcal mol^−1^). Upon excitation to *mpg‐CN, four H^+^/e^−^ pairs are transferred, forming four equivalents of mpg‐CN(H^+^/e^−^) as **I** is converted through **II**, **III,** and **IV** to **3a** (see **Figure** **4** for the energy profile). The overall energy change during those steps is −2.4 kcal mol^−1^, that is, energy difference between “**I** + 4 mpg‐CN” and “**3a** + 4 mpg‐CN(H^+^/e^−^)”. TDDFT determined energy of the mpg‐CN excited state to be 62 kcal mol^−1^ (2.69 eV), and that of the Co species +43 kcal mol^−1^ (1.86 eV). These values are consistent with that derived from UV‐vis absorption and fluorescence spectra.

**Figure 5:**
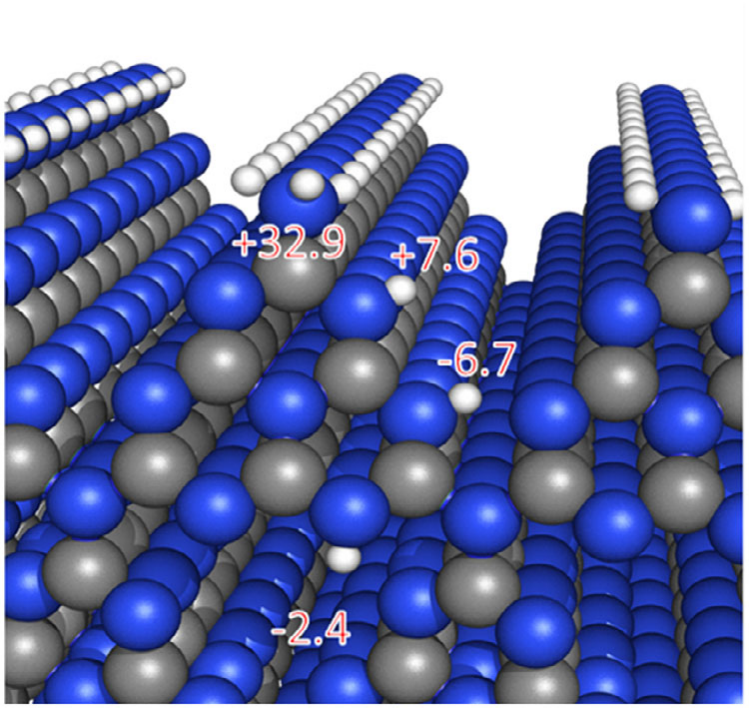
Possible sites for binding H* on the **(ac)** plane of mpg‐CN. Values represent adsorption energy (in kcal mol^−1^) relative to ½ H_2_. Negative values indicate favorable adsorption.

As discussed earlier, the uncatalyzed reaction (thermodynamic limit) has an energy change of +39.2 kcal mol^−1^. This difference, which accounts for the superior performance of mpg‐CN, is explained by a strong adsorption of **3a** (−41.9 kcal mol^−1^, i.e., energy difference between **3a** adsorbed on mpg‐CN and desorbed **3a** + mpg‐CN) and the propensity of mpg‐CN to attract hydrogen.^[^ ^38^ ^]^ Relative to H^+^ + e^−^ (which is in equilibrium with ½ H_2_ at SHE conditions), mpg‐CN(H^+^/e^−^) with H^+^ at the most favorable site has a − 0.29 eV (−6.7 kcal mol^−1^) lower energy than mpg‐CN, indicating a facilitated transfer (see **Figure** **5** for the comparison). The preferential storage of H^+^ in a “triangular pocket” of the **(ac)** surface of mpg‐CN agreewith the results of modeling obtained earlier.^[^ ^39^ ^]^

**Figure 6:**
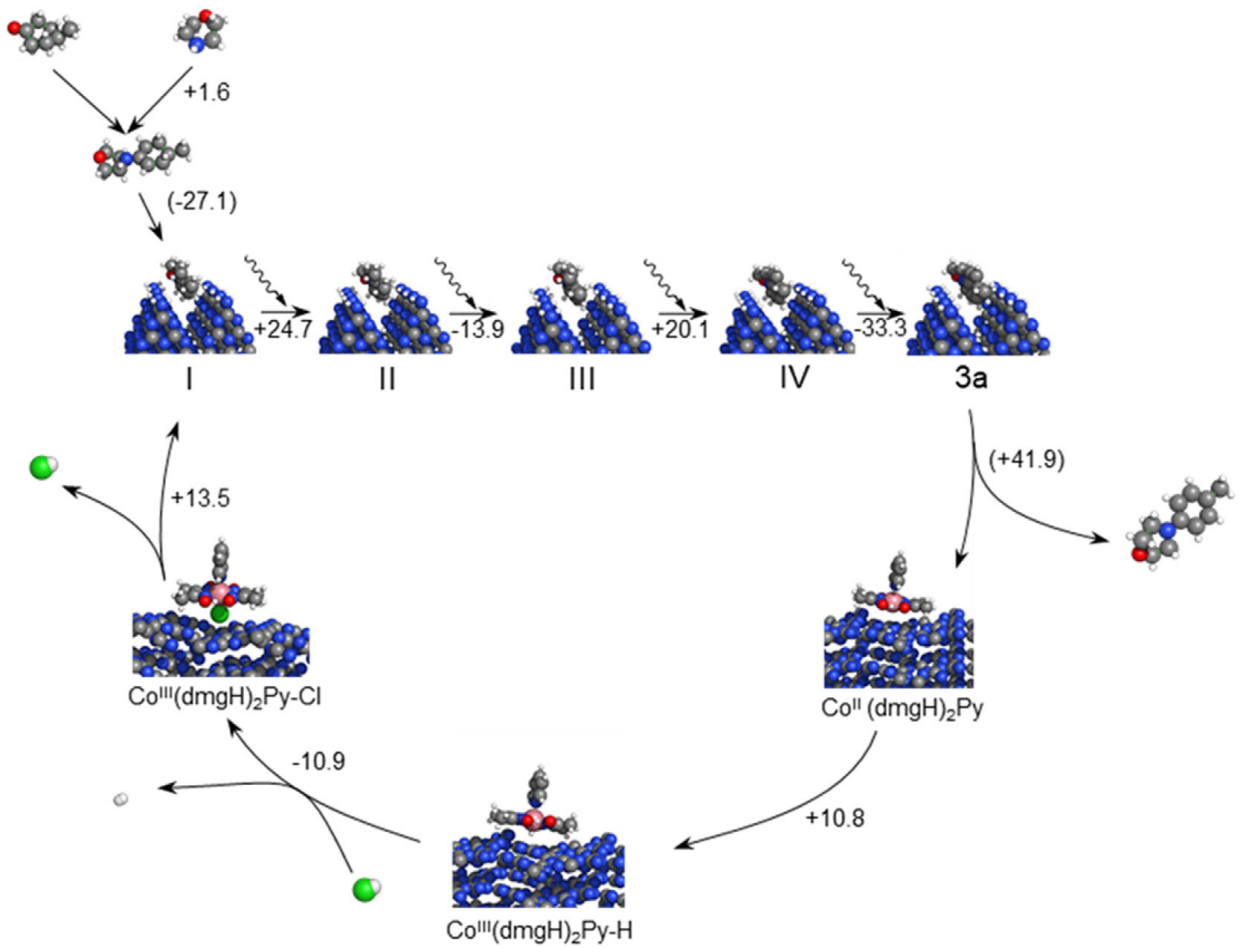
A complete reaction mechanism along with the energy differences for each reaction step (in kcal mol^−1^). Adsorption/desorption energies are shown in parentheses. The steps involving the Co‐complex transfer 2 H^+^/e^−^ and must occur twice in a cycle (see Figure 8).

Subsequently, dehydrogenation occurs as mpg‐CN(H^+^/e^−^) transfers hydrogen atoms to the Co^II^ complex (Co^II^(dmgH)_2_Py). Note that XAS measurements revealed Co(II) being persistent species. In the first step, a single hydrogen atom is transferred, forming Co^III^(dmgH)_2_PyH. In the next step, Co^III^(dmgH)_2_PyH reacts with H^+^Cl^−^, being converted to Co^III^(dmgH)_2_PyCl and releasing H_2_. The cobalt complex is recovered upon a reaction with the second equivalent of mpg‐CN(H^+^/e^−^), forming mpg‐CN, HCl, and Co^II^(dmgH)_2_Py. The whole reaction pathway is shown in **Figure** **6**.

**Figure 7:**
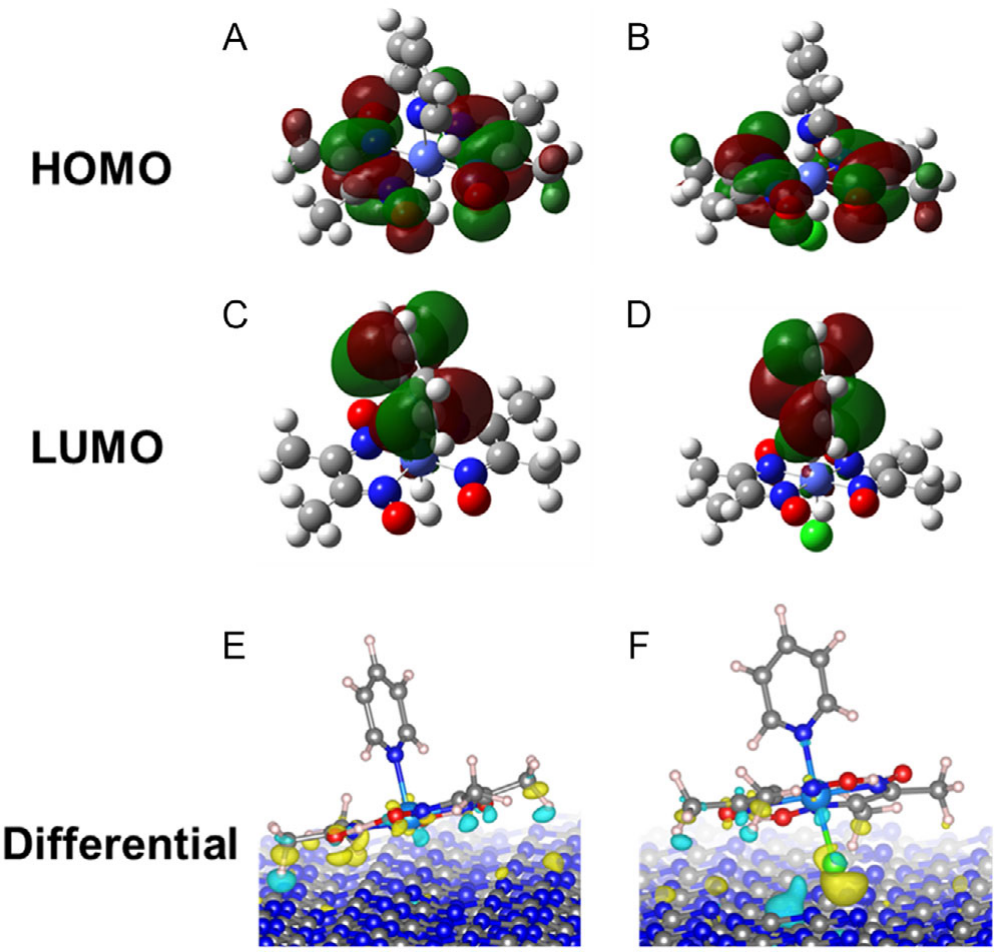
A,B) Depiction of HOMO (top), C,D) LUMO (middle) orbitals and E,F) differential charge density upon adsorption to mpg‐CN (bottom) of Co^II^(dmgH)_2_PyH (left) and Co^II^(dmgH)_2_PyCl (right).

Lastly, we studied the effect of different Co‐complexes. Co^II^(dmgH)_2_Py was modeled to get insights into the results of XAS measurements. In the unbound Co^II^(dmgH)_2_Py, Co is formally in the oxidation state of +2. The natural bond orbital (NBO6) analysis shows that Co has a net charge of 0.99 *e*_0_. Upon a H^+^/e^−^ transfer, the metal center in Co(dmgH)_2_PyH is partially reduced to 0.63 *e*_0_, while the newly transferred H retains its electron (charge 0.02 *e*_0_). Upon full oxidation, the charge on Co in the ensuing Co^III^(dmgH)_2_PyCl is 0.86 *e*_0_. In **Figure** **7**, highest occupied molecular orbital (HOMO) and lowest unoccupied molecular orbital (LUMO) are shown. This reveals two important considerations. First, formal oxidation states are rough approximations at best, which only qualitatively follow the calculated values, while the difference in the calculated charges during the reaction is much less extreme. Secondly, in Co^III^(dmgH)_2_PyCl the Co atom is (ever so slightly) *less* oxidized compared to Co^II^(dmgH)_2_Py. This difference is, however, even smaller when in contact with mpg‐CN (1.02 vs 1.09* e*_0_), where some charge transfer is also observed (*vide infra*).

Co^II^(dmgH)_2_Py and Co^III^(dmgH)_2_PyCl must approach the catalyst for an efficient hydrogen transfer. Simulations show that the cobaloxime can only approach mpg‐CN from the side along the (**ab**) facet. Their interaction energy is similar, 25.3 and 24.1 kcal mol^−1^. As shown by XAS and XPS, there is considerable charge transfer to mpg‐CN. The Bader charge of the unperturbed surface N atoms in mpg‐CN is −1.1 *e*_0_.Co^II^(dmgH)_2_Py is positioned with Co above one N atom, which has a Bader charge of −2.0 *e*_0_.On the contrary, Co^III^(dmgH)_2_PyCl is roughly between two surface N atoms, which have Bader charges of −1.81 and −1.67 *e*_0_, indicating a cumulatively larger charge transfer. This is also graphically shown in Figure 7.

**Figure 8:**
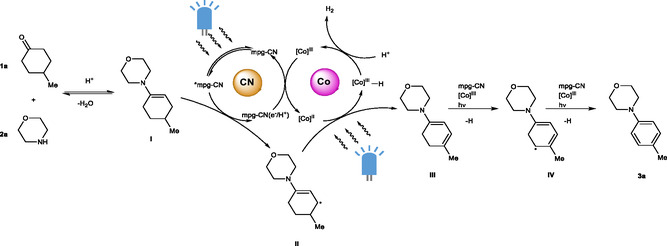
A proposed photocatalytic mechanism of **3a** synthesis by employing a combination of Co(dmgH)_2_PyCl and mpg‐CN.

Leonori and colleagues concluded involvement of two photons for each molecule of aniline formed.^[^ ^34^ ^]^ The results of modeling revealed energy barriers greater than ≈30 kcal mol^−1^ (oxidation on mpg‐CN) and greater than ≈10–20 kcal mol^−1^ (cobaloxime cycle). Given that all the experiments were conducted at ≈25–35 °C, it is unlikely that the ambient environment can provide sufficient energy to overcome the energy barriers by heat only. Therefore, involvement of more than two photons per each molecule of aniline formed is deemed to be reasonable, which is reflected in **Figure** **8**.

## 3 Conclusions

The photocatalytic synthesis of substituted anilines from carbonyl compounds and amines, a reaction of critical importance in synthetic chemistry, has traditionally relied on molecular catalysts based on rare‐earth elements, which poses sustainability challenges. This study demonstrates that merging the mesoporous graphitic carbon nitride (mpg‐CN) with cobaloxime can serve as an effective alternative to molecular catalysts in the synthesis of substituted anilines. Specifically, a combination of mpg‐CN and cobaloxime facilitates aromatization of enamine that is accompanied by evolution of hydrogen. This method efficiently couples aliphatic ketones with amines to produce substituted anilines and hydrogen in a heterogeneous manner. Mechanistic studies, supported by X‐ray spectroscopy and density functional theory (DFT) calculations, reveal charge transfer from cobaloxime to mpg‐CN in the dark and light‐induced electron transfer from mpg‐CN to cobaloxime, leading to the formation of persistent Co(II) species. This process effectively stores photon energy in the reaction products, making the energetically uphill reaction feasible. Furthermore, detailed catalyst characterization under reaction conditions confirmed the formation of a heterojunction, underscoring the efficacy of the proposed catalytic system for the synthesis of anilines using various ketones. This method not only demonstrates the potential for more sustainable and efficient production of anilines but also highlights the importance of integrating heterogeneous photocatalysts with molecular catalysts for advanced organic transformations.

## 4 Experimental Section

4.1

4.1.1

4‐Methylcyclohexanone (GC purity) was purchased from TCI, while morpholine (ReagentPlus quality), Chloro(pyridine)bis(dimethylglyoximato)cobalt(III), cyanamide, and Ludox HS‐40 were obtained from Sigma‐Aldrich at the highest commercial quality. 4‐Methylcyclohexenone, 1,4‐cyclohexanedione, and 2‐cyclohexen‐1‐one were sourced from ABCR Chemicals, also at the highest commercial quality. All chemicals were used as received without further purification.

##### Synthetic Procedures: Catalyst Preparation

mpg‐CN was prepared using the following steps: cyanamide (30 g) was mixed with an aqueous colloidal suspension of silica (75 g, LUDOX HS‐40, 40 wt%) until a clear solution was obtained. The mixture was then subjected to slow evaporation using a rotary evaporator until it formed a transparent solid. Next, the flask containing the solid was promptly placed into a water bath preheated to 80 °C and connected to a gas trap. As the solid is heated, it turned white, accompanied by the evolution of ammonia. The white solid was subsequently transferred to a crucible and heated in an oven. The temperature was increased from room temperature to 550 °C within 4 h (heating rate 2.2 °C min^−1^), followed by additional calcination at 550 °C for another 4 h under a flow of nitrogen. After cooling, the contents of the crucible were transferred to a polypropylene bottle, into which NH_4_HF_2_ (120 g) and water (500 mL) were added. The mixture was stirred for 24 h. Following filtration, the solid was washed multiple times with water and ethanol before being dried overnight in a vacuum oven. The final yield was 15.8 g.

##### Synthetic Procedures: General Procedure Dehydrogenative Synthesis of Anilines

A 5 mL vial was fitted with a magnetic stirrer and filled with mpg‐CN (20 mg), chloro(pyridine)bis(dimethylglyoximato)cobalt(III) (0.5 mg, 0.0012 mmol), and DABCO (25 mg, 0.22 mmol), all mixed thoroughly. Subsequently, 1,4‐dioxane (1 mL), 4‐methylocyclohexan‐1‐one (0.0125 mL, 0.1 mmol), morpholine (0.059 mL, 0.68 mmol), and CH_3_COOH (0.005 mL, 0.0874 mmol) were added to the mixture. The vials were sealed with screw caps equipped with a poly(tetrafluoroethylene) (PTFE) liner and degassed with Ar using a double needle technique. The reaction mixture was then irradiated for 24 h inside a blue photoreactor (*λ*_EM_ = 465 nm, *E*_EM_ = 80 mW cm^−2^). After the reaction, the catalyst was separated by centrifugation, and the solution was transferred back into the vial for analysis by GC‐MS.

##### Synthetic Procedures: General Procedure for Scale‐Up Experiment

In a three‐neck reactor equipped with a magnetic stirring bar, mpg‐CN (1.4 g), chloro(pyridine)bis(dimethylglyoximato)cobalt(III) (35 mg, 0.084 mmol), and DABCO (1.75 g, 15.7 mmol) were placed and thoroughly mixed. Subsequently, 1,4‐dioxane (70 mL), 4‐methylocyclohexan‐1‐one (0.875 mL, 7 mmol), morpholine (4.13 mL, 47.6 mmol), and CH_3_COOH (0.35 mL, 6.125 mmol) were added to the mixture. The reactor was then degassed with Ar, and the temperature sensor was immersed while the gas collector was connected to the reactor neck. The reaction mixture was irradiated for 24 h inside a blue photoreactor. After the 24‐hour reaction period, the reaction mixture (1 mL) was extracted, the catalyst was separated by centrifugation, and the solution was analyzed by GC‐MS. The reaction was considered complete when all starting materials were consumed. The catalyst was further separated from the reaction mixture by centrifugation. Subsequent steps involved the isolation of the reaction product. Firstly, the dioxane was removed using a rotary evaporator. Then, dichloromethane was used to dissolve the remaining liquid. Next, the solution was washed successively with sodium bicarbonate, water, and brine. Finally, the solvent was removed using a rotary evaporator. Purification of the crude liquid was carried out by hand column chromatography using ethyl acetate/hexane as eluents on silica gel.

##### Synthetic Procedures: General Procedure for Catalyst Stability Tests

The reaction was performed in a 5 mL vial as previously described. In the first experiment, the recovered catalyst was washed with ethanol, dried, and then reused for another reaction. After 24 h of irradiation, the catalyst was recovered by centrifugation, washed with ethanol, and dried at 60 °C in a vacuum oven. After each catalytic run, the reaction mixture was collected and analyzed by GC‐MS.

In the second experiment, the chloro(pyridine)bis(dimethylglyoximato)cobalt(III) was replenished. After the first catalytic run, the catalyst was recovered, washed with ethanol and dried at 60 °C. For the second reaction cycle, the recovered catalyst was combined with an additional chloro(pyridine)bis(dimethylglyoximato)cobalt(III) (0.5 mg, 0.0012 mmol) and the standard reaction mixture. After 24 h of irradiation, the catalyst was again recovered, washed, and dried. This procedure was repeated for a third cycle with the addition of a fresh portion of the cobaloxime (0.5 mg, 0.0012 mmol). After each catalytic run, the reaction mixture was collected and analyzed by GC‐MS.

##### Synthetic Procedures: General Procedure for Reactions with Different Ketones in 0.1 mmol Scale

A 5 mL vial was fitted with a magnetic stirrer and filled with mpg‐CN (20 mg), chloro(pyridine)bis(dimethylglyoximato)cobalt(III) (0.5 mg, 0.0012 mmol), and DABCO (25 mg, 0.22 mmol), all mixed thoroughly. Subsequently, 1,4‐dioxane (1 mL), chosen ketone (0.1 mmol), morpholine (0.059 mL, 0.68 mmol), and CH_3_COOH (0.005 mL, 0.0875 mmol) were added to the mixture. The vials were sealed with screw caps equipped with a PTFE liner and degassed with Ar using a double needle technique. The reaction mixture was then irradiated for 24 h inside a blue photoreactor (*λ*_EM_ = 465 nm, *E*_EM_ = 80 mW cm^−2^). After the reaction, the catalyst was separated by centrifugation, and the solution was transferred back into the vial for analysis by GC‐MS.

##### Synthetic Procedures: General Procedure for Reactions with Different Ketones in 5 mmol Scale

In a three‐neck reactor with a magnetic stirring bar, mpg‐CN (1 g), chloro(pyridine)bis(dimethylglyoximato)cobalt(III) (25 mg, 0.06 mmol), and DABCO (1.25 g, 11 mmol), was combined and mixed. Then, 1,4‐dioxane (50 mL), along with 4‐metoxycyclohexan‐1‐one (A) or 1,4‐cyclohexanedione (B) or and 2‐cyclohexen‐1‐one (C) (5 mmol each), morpholine (2.95 mL, 34 mmol), and CH_3_COOH (0.250 mL, 4.375 mmol) were added. After degassing the reactor with Ar, the temperature sensor and connected the gas collector were submerged. The reaction mixture underwent 24 h of irradiation inside a blue photoreactor. After that time, the reaction mixture (1 mL) was extracted, catalyst was separated by the centrifugation, and the solution was analyzed by GC‐MS. The reaction was considered finished when all starting materials were used up. We then isolated the catalyst from the reaction mixture by centrifugation. Next steps included removing dioxane with a rotary evaporator, dissolving the remaining liquid in dichloromethane, washing the solution with sodium bicarbonate, water, and brine, and finally removing the solvent with a rotary evaporator. To purify the crude liquid, we conducted hand column chromatography on silica gel using ethyl acetate/hexane as eluents.

##### Physicochemical Characterization of Catalyst

XAS operando experiments in the soft X‐ray regime were performed at the APE‐HE beamline of the ELETTRA synchrotron radiation facility in Trieste, Italy, at the N K‐ and Co L2,3‐edges. The experiments were carried out in flowing He at 50 standard cubic centimeter per minute (SCCM), in the operando XAS cell at room temperature, in dark conditions and under illumination with a 40 mW laser at 405 nm. The crystallinity of the materials was determined by powder X‐ray diffraction (XRD) using Rigaku SmartLab (Japan, Cu Kα, 0.154 nm). Thermo Scientific Nicolet iD7 spectrometer was used as a Fourier‐transform infrared (FTIR) spectrometer. Physisorption measurements were performed on a Quantachrome Quadrasorb SI at 77 K for N_2_. The specific surface areas were calculated by applying the Brunauer–Emmett–Teller (BET) model to adsorption isotherms for 0.05 < p/p_0_ < 0.3 using the QuadraWin 5.11 software package. Samples were degassed overnight before the measurements. Scanning electron microscopy (SEM) imaging was performed using the Zeiss LEO 1550‐Gemini (Germany) system with acceleration voltage of 10 kV. An Oxford Instruments X‐MAX 80 mm^2^ detector was used to collect the energy‐dispersive X‐ray (EDX) data. Fluorescence spectra were recorded on Jasco FP‐830 instrument (Japan). The excitation wavelength was set at 375 nm. Optical absorbance spectra of powders were measured on a Shimadzu UV 2600 equipped with an integrating sphere. Electron paramagnetic resonance (EPR) study was conducted on Bruker EMXnano benchtop X‐Band EPR spectrometer. The following settings have been used for all spectra acquisition unless other is specified: center field 3448.05 g, sweep width 200 g, receiver gain 50 db, modulation amplitude 1.000 g, number of scans 10, and microwave attenuation 20 dB. Sample were placed and flame‐sealed in EPR capillaries (IntraMark, volume 50 μL, ID 0.86 mm), inside the EPR tube (ID 3 mm, OD 4 mm, length 250 mm). Photoluminescence/excitation maps were recorded by Horiba FluoroMax‐4, integration time 0.2, and slits aperture of 2. Mass spectral data were obtained using Agilent GC 8890 gas chromatograph, equipped with HP‐5MS column (inner diameter = 0.25 mm, length = 30 m, and film = 0.25 μm), coupled with Agilent GC/MSD 5977B mass spectrometer (electron ionization). ^1^H NMR spectra were recorded on Agilent 400 MHz Bruker.

##### Computational Methods

Thermodynamic calculations were performed at the DFT level using Gaussian 16. The wB97XD functional^[^ ^40^ ^]^ was chosen on account of its favorable performance for main‐group elements and organo‐metallics and inclusion of the D2 dispersion interaction by Grimme. Geometrical optimizations were done with the 6–31 + G(d,p) basis set and final energies were obtained as single‐pass calculations with the 6–311++G(d,p) basis set. In all instances, the SMD variation of the IEFPCM solvent was used with the default settings for dioxane.^[^ ^41^ ^]^ Vibrational analysis was performed to confirm that no imaginary frequencies were present in stable structures. Vibrational, rotational, and translational contributions to the free energy were accounted for. Gibbs free energies were computed at 1 atm and 298.15 K.^[^ ^42^ ^]^ These calculations were used to study the thermodynamics and equilibria of the reactions involved in the process.

A combined H^+^/e^−^ transfer from the substrate to mpg‐CN was modeled with a plane‐wave DFT approach, as implemented in VASP 6.3.1, due to the periodic structure of mpg‐CN. It was modeled as a perfectly condensed graphitic carbon nitride.^[^ ^43^ ^]^ It assumes an undulating structure with a computationally optimized unit cell *a* = 7.00, *b* = 12.12, *c* = 7.00 Å, and C_24_N_32_ composition. Consistent with previous works, a slightly off‐set AA structure, where the layers are shifted by 12% and 7% in the cardinal directions, is most stable. The structure is not planar but assume a zigzag‐type geometry, consistent with.^[^ ^44^ ^]^ The active site of mpg‐CN is the (ac) edge plane, which was modeled in a 2 × 2 supercell with hydrogen‐terminated dangling nitrogen atoms (−NH_2_). Along the (ac) edge, mpg forms pores, where **I** (4‐(4‐methylcyclohexen‐1‐yl)morpholine) binds to and undergoes a stepwise oxidation as H^+^ + e^−^ is transferred to *mpg‐CN under illumination. The re‐oxidation of mpg‐CN(H^+^/e^−^), however, can only occur at the edges (ab), since the (Co(dmgH)_2_PyCl) complex does not fit into the pores on (ac). A RPBE functional^[^ ^45^ ^]^ was used with an energy cutoff of 500 eV. A Gaussian smearing of 0.001 eV was used to improve the electronic convergence. Spin‐polarized calculations were used where required and the Grimme D3 dispersion correction was turned on.^[^ ^46^ ^]^ Due to the size of the supercell, a single point (gamma) sampling in the Brillouin zone sufficed. Whenever multiple possibilities existed, all possible adsorption sites were considered, and the lowest‐lying energies are reported universally.

## Conflict of Interest

The authors declare no conflict of interest.

## Supporting information

Supplementary Material

## Data Availability

The data that support the findings of this study are available from the corresponding author upon reasonable request.
