# Supplementary material for: Merging Heterogeneous Graphitic Carbon Nitride Photocatalysis with Cobaloxime Catalysis in Uphill Dehydrogenative Synthesis of Anilines
Source: ChemSusChem. 2025 Apr 9;18(12):e202402439. doi: 10.1002/cssc.202402439 (PMC12175032; doi:10.1002/cssc.202402439)
Supplement: Supplementary file 1 — Supplementary Material [file CSSC-18-e202402439-s001.pdf]

Supplementary Information

Merging Heterogeneous Graphitic Carbon Nitride Photocatalysis with Cobaloxime Catalysis in Uphill Dehydrogenative Synthesis of Anilines

Sonia Zoltowska,[‡] Stefano Mazzanti,[‡] Sara Stolfi, Jingsan Xu, Matej Huš, Ana Oberlintner, Matic Pavlin, Paolo Ghigna, Blaž Likozar, Piero Torelli, Luca Braglia, Davide Ravelli, Maurizio Fagnoni, Iker Agirrezabal-Telleria, Markus Antonietti, Paolo Giusto, Oleksandr Savateev*

Supplementary note 1: Reusability test and analysis of spent catalyst

To evaluate the reusability and durability of the investigated catalytic system, a series of recycling studies were performed. The objective was to determine if the system could maintain its efficiency over multiple reaction cycles and to compare the yields when the cobaloxime was replenished versus when the catalytic system was used without cobaloxime replenishment.

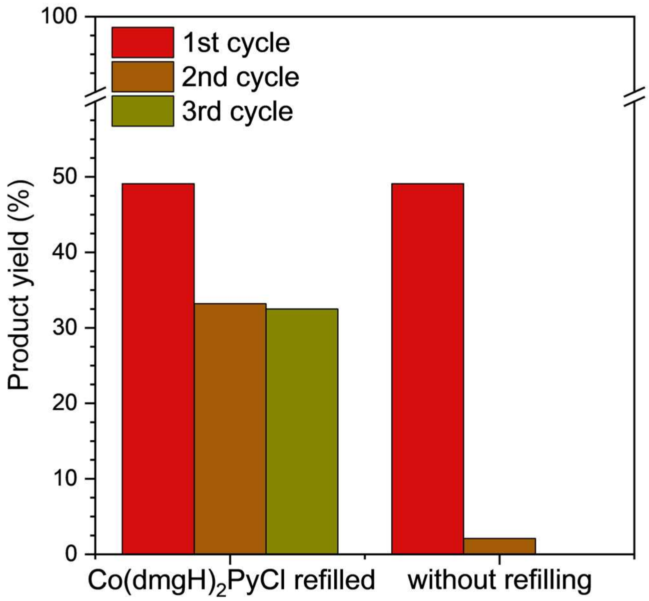

Fig. S1. Catalyst recycling study.

What is striking about the data is that the mpg-CN/Co(dmgH)$_2$PyCl system shows a significant drop in activity upon reuse without replenishment of the cobaloxime, resulting in only trace amounts of products. This indicates that both materials work separately in the catalytic cycle: mpg-CN is responsible for the oxidation of the enamine in the first step, while Co(dmgH)$_2$PyCl is crucial for completing the cycle by performing the dehydrogenation. Interestingly, when Co(dmgH)$_2$PyCl is replenished, only a slight loss of activity is observed, which can be attributed to potential deactivation caused by the accumulation of reaction by-products that block active sites or structural changes in the mpg-structure after long irradiation, as it was suggested by XAS analysis.

To evaluate in detail the pathway of catalyst changes, the spent catalyst was analysed using bulk (XRD) and optical techniques (UV-Vis, FTIR and XPS).

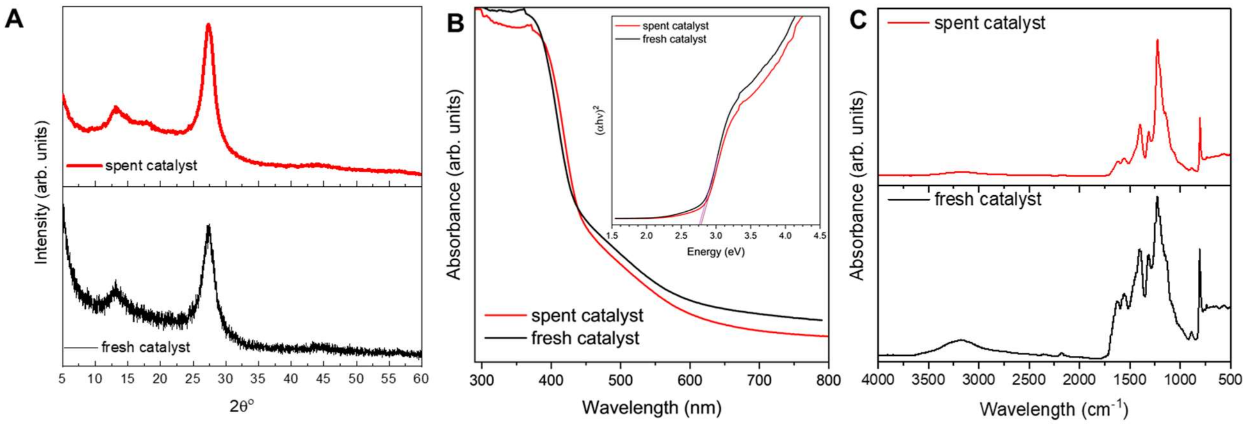

Fig. S2. (A) XRD pattern of spent Co(dmgH)$_2$PyCl/mpg-CN (upper figure) and fresh photocatalyst (bottom figure), (B) DRUV-Vis absorption spectrum with Tauc plot, (C) FTIR spectrum of spent Co(dmgH)$_2$PyCl/mpg-CN (upper figure) and fresh photocatalyst (bottom figure).

The XRD pattern of the spent catalyst exhibits significant changes compared to that of freshly prepared mpgCN. Specifically, the (100) diffraction peak shows decreased intensity and broadening, indicating a disruption in the periodic arrangement of heptazine units. This suggests a loss of structural order, likely due to prolonged light exposure. Additionally, the peak broadening may reflect increased disorder within the mpgCN framework, possibly resulting from surface oxidation. A comparison of the UV-Vis spectra reveals noticeable changes. The spent catalyst (red line) shows a decrease in absorption intensity, particularly in the UV region (~300–450 nm), accompanied by a slight shift in the absorption edge. This can be attributed to prolonged exposure to blue light and reaction conditions, which may induce partial degradation of mpgCN or minor structural modifications, leading to a reduction in UV-range absorption. While visible-light absorption (above 450 nm) remains relatively unchanged, a slight band gap widening is observed. This could result from surface oxidation or minor decomposition of Co(dmgH)$_2$PyCl during the reaction cycle, which alters electronic transitions. Additionally, reaction by products may accumulate on the catalyst surface, blocking active sites and further influencing electronic transitions. Consequently, reintroducing fresh Co(dmgH)$_2$PyCl may be necessary to restore catalytic performance, as demonstrated above. The FTIR spectra indicate that the spent catalyst undergoes surface modifications, evidenced by peak broadening and intensity reduction in the 1200–1600 cm^{-1} region. Similarly, the diminished intensity of the characteristic peak associated with triazine ring breathing modes suggests partial degradation of the mpgCN backbone due to prolonged light irradiation. Additionally, reaction products and intermediates may adsorb onto the catalyst surface, altering the FTIR spectrum. Further evidences about the catalyst alteration upon reaction was depicted using XPS spectroscopy (Fig. S3).

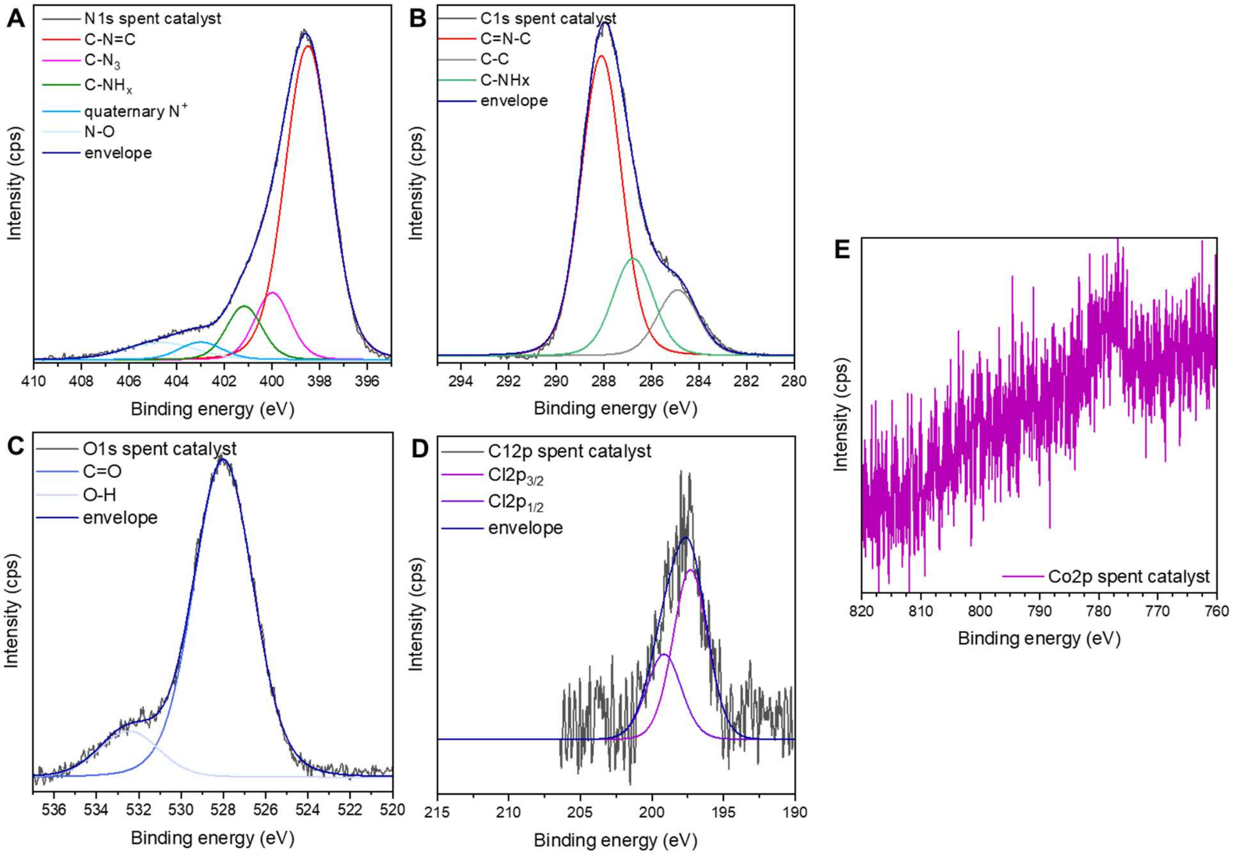

Fig. S3. XPS spectra of $Co(dmgH)_2PyCl$/mpg-CN spent catalyst: N 1s (A), C 1s (B), O 1s (c), Cl 2p (D) and Co 2p (E).

The noticeable broadening of peaks in the N 1s spectrum—for instance, the C–N=C peak widening from 1.3 eV in freshly prepared $Co(dmgH)_2PyCl$/mpgCN to 2.7 eV in the spent catalyst—confirms oxidation and increased structural disorder in the mpgCN backbone due to prolonged light exposure. Additionally, the rise in N–O and quaternary N^+ species in the spent catalyst suggests photoinduced oxidation of nitrogen sites. However, contributions from the π–π shake-up satellite at ~402.9 eV and an additional component at ~404.6 eV cannot be ruled out[1, 2]. Significant changes are also observed in the C 1s spectrum, where an increase in the $C–NH_x$ peak suggests enhanced surface modifications or the incorporation of additional nitrogen-containing species. The overall peak broadening (increased FWHM) in the spent catalyst spectrum indicates greater structural heterogeneity, likely due to oxidation-induced surface defects or partial degradation of mpgCN. The O 1s spectra comparison clearly shows fewer distinct oxygen species, with broadening of the main O 1s peak, confirming greater oxygen-related heterogeneity. The increased presence of carbonyl and hydroxyl moieties suggests partial reaction-induced modification of the catalyst upon prolonged light exposure, ultimately reducing its stability. Moreover, the Cl 2p spectrum of the spent catalyst reveals a decrease in peak intensity, suggesting partial loss of chloride ligands, likely due to Cl^- leaching from the $Co(dmgH)_2PyCl$ complex during the photocatalytic reaction. The peak broadening indicates that the remaining chlorine species exist in a more disordered state, possibly as adsorbed or weakly coordinated species. The low signal-to-noise ratio in the Co 2p spectrum confirms that only a small

amount of the cobalt complex remains on the photocatalyst surface after the catalytic cycle. Consequently, reintroducing fresh Co(dmgH)$_2$PyCl improved catalyst longevity.

In conclusion, prolonged light exposure leads to photocatalyst degradation, primarily through heptazine framework disruption, which results in a loss of active catalytic sites and diminished performance.

Supplementary note 2: Scale-up and expanding the reaction scope

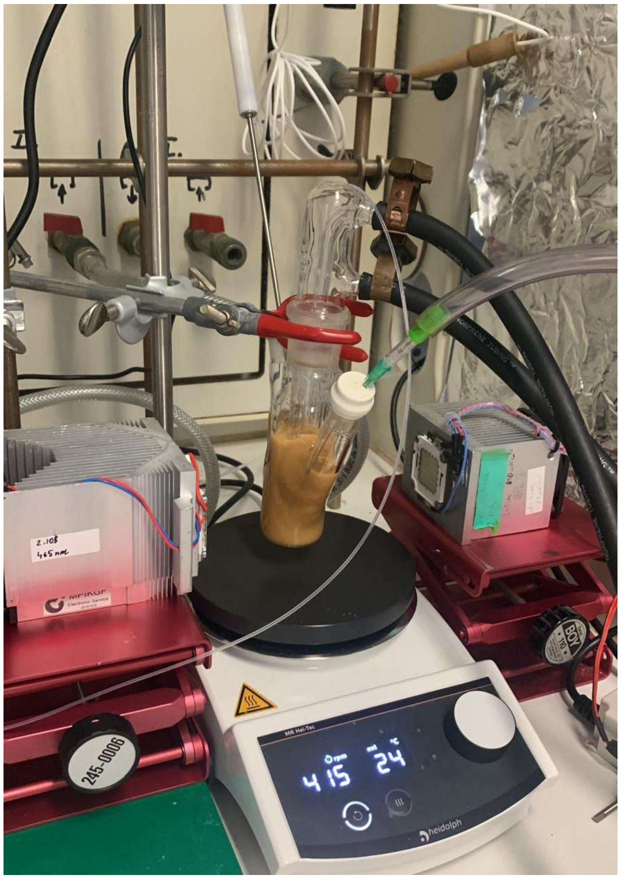

Fig. S4. Typical set-up for upscaling of the photocatalytic system.

Generation of H_2 and aniline ratio was confirmed in the experiment conducted on 7 mmol scale of 4-methylcyclohexanone resulting in formation of aniline with 35% yield (**Fig. S4**).

To evaluate the tolerance of the catalytic system, four different ketones were selected and subjected to optimal reaction conditions in a qualitative manner (**Fig. S5**).

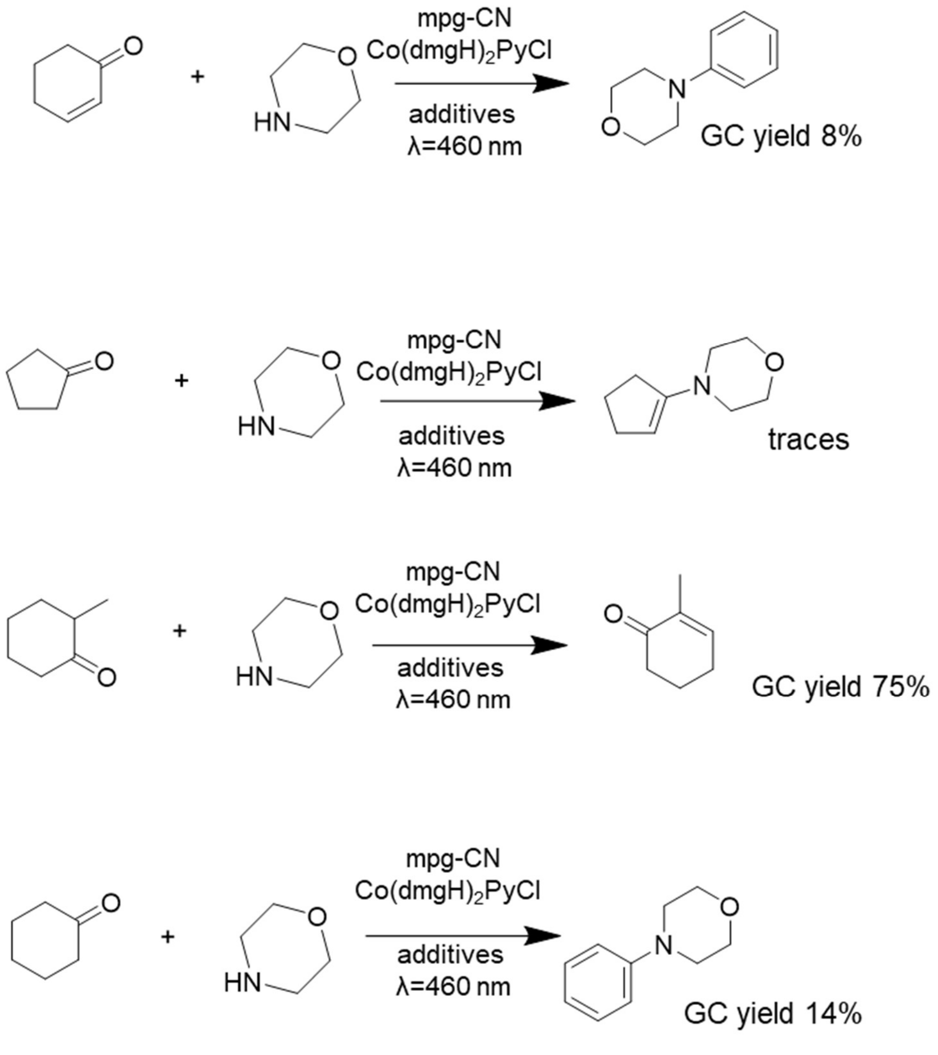

Fig. S5. Schematic representation of qualitative reactions of morpholine with different ketones[a].

[a]Applied conditions: Reaction conditions: ketone (0.1 mmol, 0.0125 mL), morpholine (0.68 mmol, 0.059 mL), mpg-CN 20 mg, $Co(dmgH)_2PyCl$ (0.0012 mmol, 0.5 mg), DABCO (0.22 mmol, 25 mg), glacial AcOH (0.0874 mmol, 0.005 mL), dioxane (1 mL), blue LEDs (λ_{max} = 460 nm, 80 mW cm^{-2}). GC yields were measured using GC-FID with 1-octadecene as internal standard.

As a result, cyclohexanone and cyclohex-2-en-1-one successfully underwent the desired transformation to produce the corresponding aniline product. These results suggest that the catalytic system is effective with both saturated and unsaturated cyclic ketones. On the other hand, 2-methylcyclohexanone did not react under the same conditions, likely due to the steric hindrance introduced by the methyl group at the 2-position. The reaction with cyclopentanone led to the formation of an enamine product rather than the expected aromatic compound. This outcome can be attributed to the fact that the aromatization of cyclopentanone would necessitate the formation of a cyclopentadienide anion—a highly reactive and

unstable species. Consequently, the reaction halts at the enamine stage, as further progression is thermodynamically unfavourable.

In next step, 3 different ketones were chosen to perform a quantitative study with hydrogen collection (**Table S1**).

Table S1. Yields of coupling products and H_2 on 5 mmol scale.[a]

Substrate	Structure of main product	GC yield of main product[b]	Evolved gas[c,d]
4-methylcyclohexenone	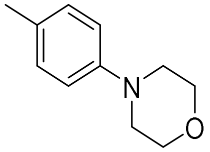	1 mmol (20 %)	2.9 mmol[c]<br>4.4 mmol[d]
1,4-cyclohexanedione	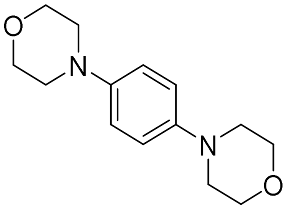	0.9 mmol (18 %)	2.2 mmol[c]<br>3.2 mmol[d]
2-cyclohexen-1-one	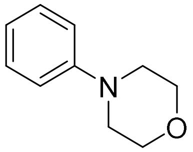	1.3 mmol (27 %)	3.3 mmol[c]<br>6.1 mmol[d]

[a] Reaction conditions: ketone (5 mmol), morpholine (34 mmol), mpg-CN (100 mg), $Co(dmgH)_2PyCl$ (0.06 mmol, 25 mg), DABCO (11 mmol, 125 mg), glacial AcOH (4.37 mmol, 0.25 mL), dioxane (50 mL), blue LEDs (λ_{max} = 460 nm, 80 mW cm^{-2}).

[b] GC yield measured after 24 hours of reaction.

H_2 was collected after 24[c] and 48[d] hours of reaction.

Supplementary note 3: Catalyst characterization

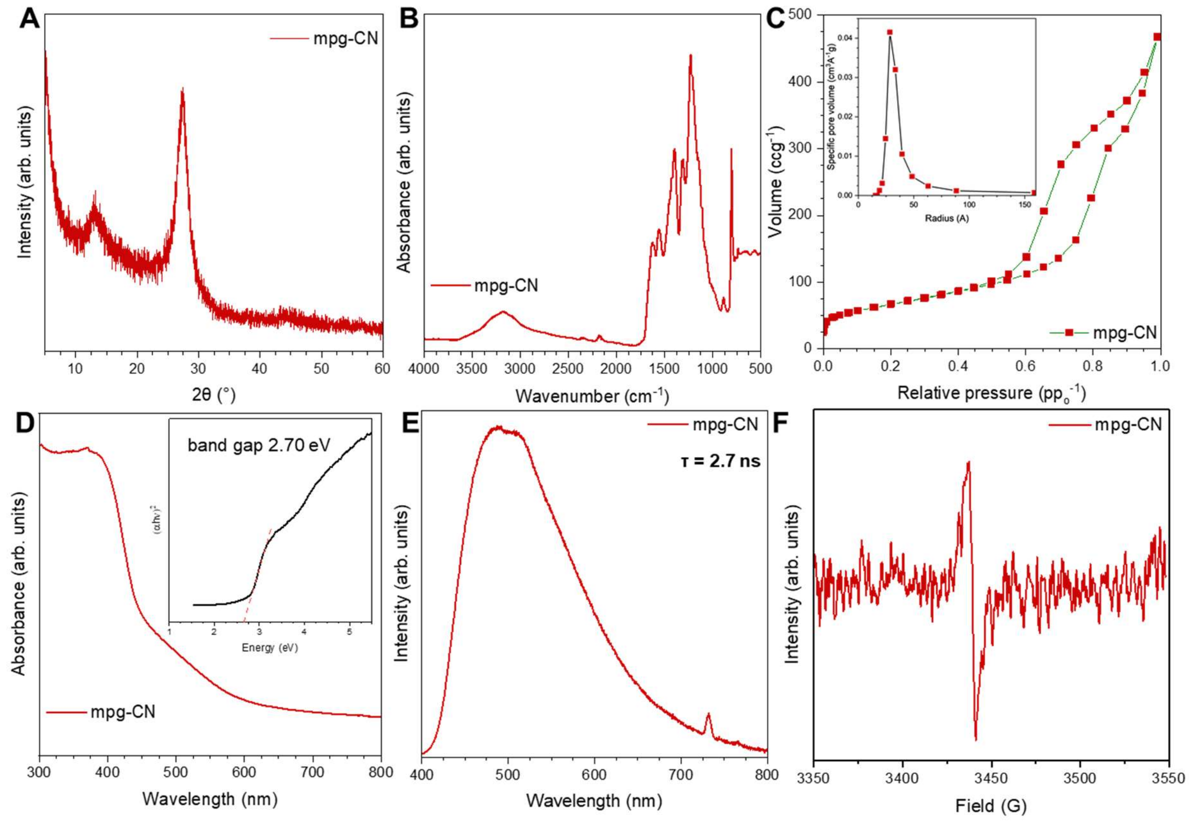

Fig. S6. XRD pattern of mpg-CN (A), FTIR spectrum of mpg-CN (B), N_2 sorption isotherms recorded at 77 K (C) DRUV-Vis absorption spectrum with Tauc-plot (D), Steady-state PL spectrum of mpg-CN with 2nd order excitation light diffraction (E), EPR spectrum of mpg-CN (F).

The mpg-CN structure and physicochemical properties are in agreement with previously reported data[3] [4]As confirmed by the XRD pattern, mpg-CN is an amorphous material characterized by two characteristic diffraction features (Fig. S6A). The first peak, at 13°, corresponds to a calculated interlayer distance 0.68 nm, is characteristic for the heptazine units, corresponds to the (100) diffraction plane. The second, more pronounced feature at 27° is associated with the (002) diffraction plane of interlayer π–π stacking of the heptazine rings, with the corresponding distance 0.33 nm. The Fourier-transform infrared spectrum of mpg-CN (Fig. S6B) exhibits typical features characteristic of carbon nitride, as well described in the literature[5, 6]. The broad band located at 3185 cm^{-1} is attributed to the stretching vibration of N-H bonds, while bending vibrations of primary and secondary amine N-H bonds are observed at 1624 and 1561 cm^{-1}, respectively. Their presence may suggest the existence of non-condensed amino groups on the surface of the material. The peak at 2183 cm^{-1} can be attributed to the nitrile stretching vibrations, and a broad band between 1650-1000 cm^{-1} is associated with heptazine stretching vibrations. The characteristic sharp band located at 805 cm^{-1} corresponds to the breathing mode of triazine ring units. Bands presented at 1311 and 1229 cm^{-1} are assigned to C-O stretching vibrations.

The surface area of mpg-CN was calculated from N_2 sorption curves using the Brunauer–Emmett–Teller (BET) method (Fig. S6C). The BET surface area was 237 $m^2\ g^{-1}$ with isotherms of type IV, characteristic of mesoporous materials, with hysteresis presenting the H3-type loop. The isotherm shows a more gradual

increase in adsorbed volume with less pronounced steepness at higher relative pressures, suggesting a mix of micro- and mesopores. The pore size distribution was measured by the Barrett–Joyner–Halenda (BJH) method, indicates the presence of mesopores with a range value between 2 and 6 nm, the broad shape of the peak suggesting the presence of micropores in the overall porous structure. In the DRS-UV-Vis spectrum (Fig. S6D), the absorption maximum around 395 nm was observed, which is correlated to the π-π* transitions typical of conjugated ring systems. In addition, a less intense feature at 500 nm corresponds to n-π* transitions of N- lone electron pairs on the edge N-atoms of heptazine rings. The room-temperature photoluminescence (PL) spectrum, excited by a UV laser (365 nm), shows a broad peak near 505 nm assigned to radiative recombination of charge carriers (Fig. S6E). The EPR spectrum of the mpg-CN (Fig. S6F) exhibits a single Lorentzian line centred at a g-value of 2.001, demonstrating a well-established carbon nitride semiconductor structure. The Lorentzian line of the catalyst has been attributed to the unpaired electron of the π-bonded aromatic rings.

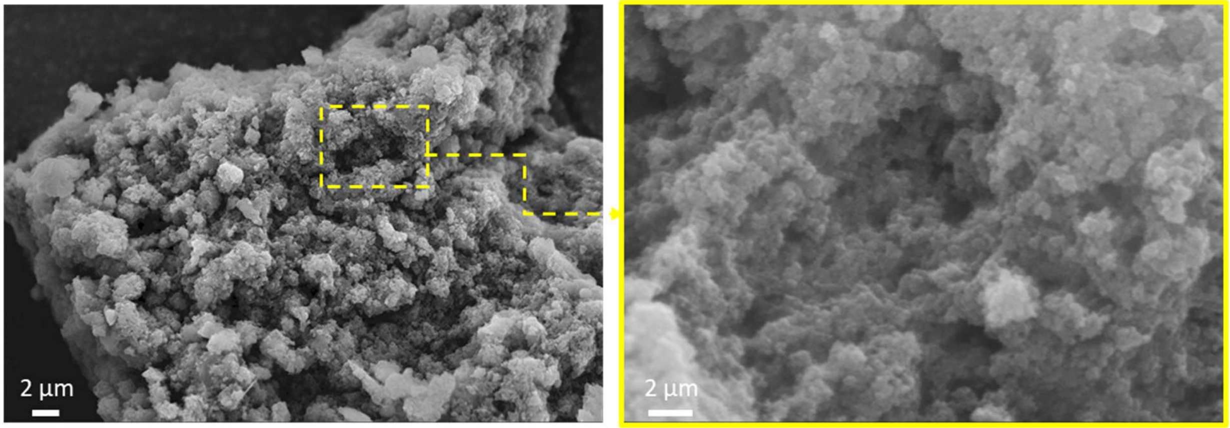

Fig. S7. SEM images of mpg-CN-237 catalyst.

Scanning electron microscopy was used to study morphology of mpg-CN particles. Microscopic images (Fig. S7) shows agglomerated round-shaped particles with the diameter in the range from few hundred nanometres to few micrometres.

Supplementary note 4: Catalyst characterization $Co(dmgH)_2PyCl$/mpg-CN

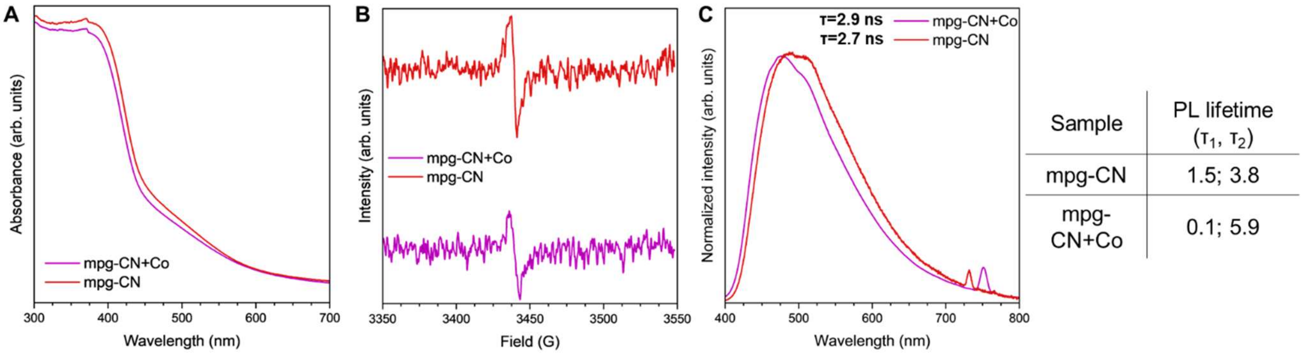

Sample	PL lifetime (τ_1, τ_2)
mpg-CN	1.5; 3.8
mpg-CN+Co	0.1; 5.9

Fig. S8. DRUV-Vis absorption spectrum with Tauc-plot (A), Steady-state PL spectrum of mpg-CN with 2nd order excitation light diffraction with photoluminescence lifetimes (B), EPR spectrum of mpg-CN+$Co(dmgH)_2PyCl$ (C).

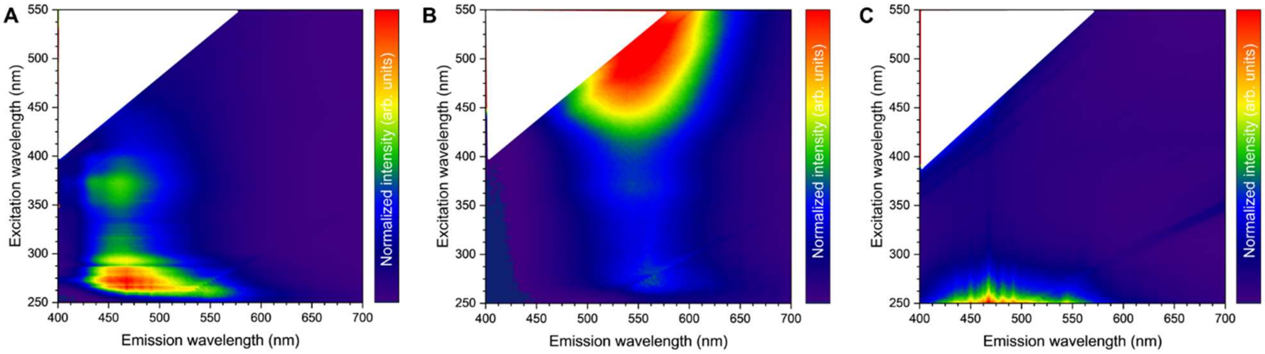

Fig. S9. Contour plot of photoexcitation versus photoluminescence spectra of mpg-CN (A) $Co(dmgH)_2PyCl$/mpg-CN (B) and $Co(dmgH)_2PyCl$ (C).

The optical characterization of the material obtained after deposition of $Co(dmgH)_2PyCl$ (2.4 wt%) on mpg-CN showed negligible changes in the electronic band structure of the resulting $Co(dmgH)_2PyCl$/mpg-CN material (Fig. S8A). Interestingly, the EPR spectra (Fig. S8B) revealed the broadened and more elaborated signal of $Co(dmgH)_2PyCl$/mpg-CN, suggesting that the complex deposition leads to the introduction of new cobalt-related paramagnetic species or the interaction of cobalt with existing ones, creating a different magnetic environment compared to the bare mpg-CN material. The steady-state PL spectra indicate that the addition of $Co(dmgH)_2PyCl$ significantly affects the photophysical properties of the material. The significant shortening of the initial lifetime could be associated with a fast, non-radiative decay pathway. On the other hand, the longer second lifetime recorded for $Co(dmgH)_2PyCl$/mpg-CN suggests the introduction or stabilization of defect states in mpg-CN that take longer to recombine and emit light. The overall short fluorescence lifetime can be attributed to charge transfer to $Co(dmgH)_2PyCl$, which enhances nonradiative relaxation.

The contour plots of photoexcitation versus photoluminescence spectra (Fig. S9) support these findings, where $Co(dmgH)_2PyCl$/mpg-CN shows a broader and more intense emission profile, especially at higher

excitation wavelengths, compared to the more confined emission of pure mpg-CN. Taken together, these results suggest that cobalt incorporation significantly alters the photophysical properties of mpg-CN, introducing new pathways for energy dissipation and affecting the luminescence behavior of the material, suggesting the formation of a heterojunction.

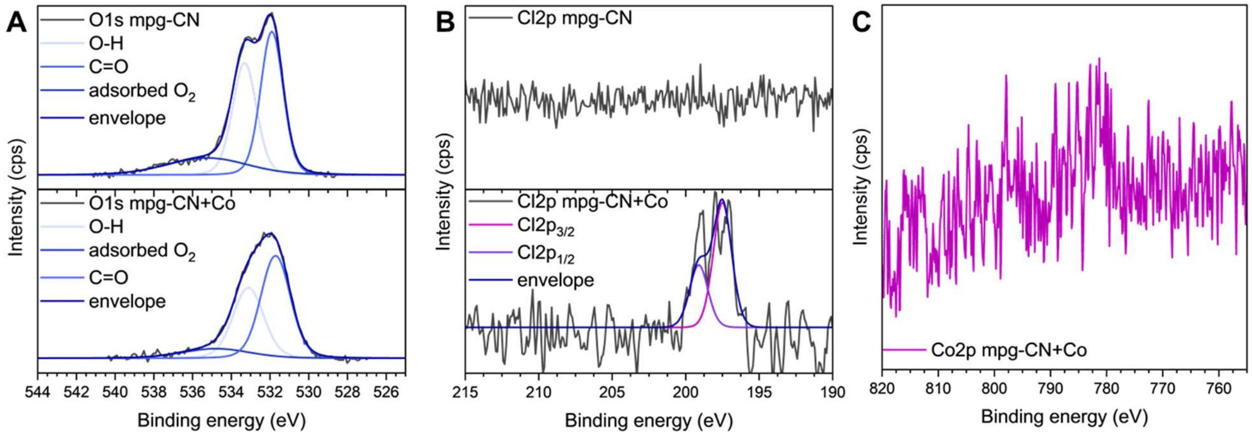

Fig. S10. XPS O 1s (A), Cl 2p (B) and Co 2p spectra of $Co(dmgH)_2PyCl$/mpg-CN.

Oxygen in mpg-CN originates from adsorbed water and partial hydroxylation of carbon nitride, i.e., substitution of a fraction of NH_2-groups with HO-groups during template removal in aqueous solution of $(NH_4)HF_2$. While mpg-CN is free of chlorine, Cl 2p spectrum of $Co(dmgH)_2PyCl$/mpg-CN revealed presence of Cl. Signal-to-noise ratio in Co 2p spectrum is low confirming the presence of minor quantities of cobalt complex in the reaction mixture (Fig. S10).

Supplementary note 5: Catalyst comparison

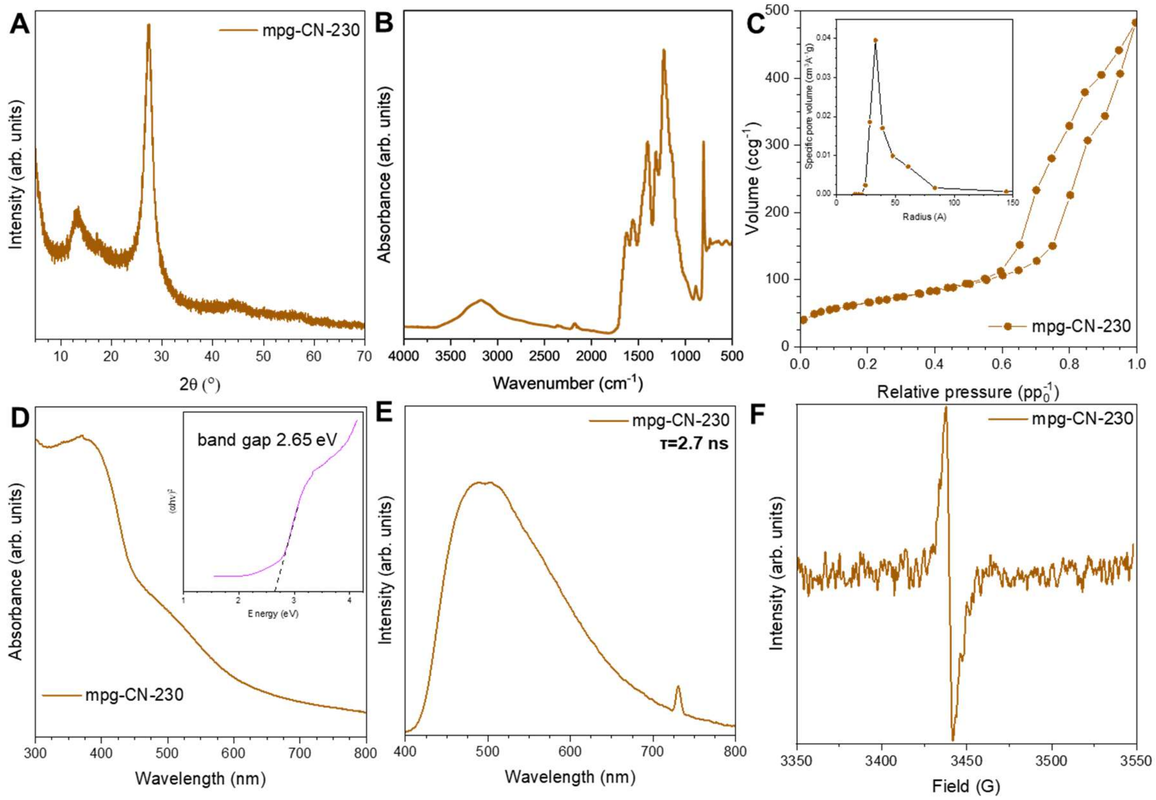

Fig. S11. XRD pattern of mpg-CN-230 (A), FTIR spectrum of mpg-CN-230 (B), N_2 sorption isotherms recorded at 77 K with pore size distribution (C) DRUV-Vis absorption spectrum with Tauc-plot (D), Steady-state PL spectrum of mpg-CN with 2nd order excitation light diffraction (E), EPR spectrum of mpg-CN-230 (F).

During the evaluation of the catalytic system, we tested a mesoporous carbon nitride material with a surface area of 230 $m^2\ g^{-1}$. The mpg-CN-230 is also an amorphous material with two characteristic diffraction features (Fig. S11A). Notably, the peaks for mpg-CN-230 are sharper and more defined compared to those for mpg-CN-237, indicating a higher degree of crystallinity or a more ordered structure in mpg-CN-230. The intensity of the peak at 27° 2θ for mpg-CN-230 is significantly higher than that of mpg-CN-237, indicating a more prevalent π-π interlayer stacking in mpg-CN-230. The pore size distribution in mpg-CN-230 ranges from 2 to 10 nm, with a significant peak around 5 nm (Fig. S11C). The distribution also shows some larger pores extending beyond 10 nm. Therefore, it should be noted that mpg-CN-230 has a more pronounced mesoporous structure with a higher volume of larger pores centred around 5 nm, while mpg-CN-237 has a broader range of pore sizes with a higher proportion of smaller mesopores around 4 nm and micropores. Spectroscopic characterization of mpg-CN-230 shows features characteristic of carbon nitride (Fig. S11B,D-F)[4, 6, 7]. In particular, the UV-Vis absorption spectra suggest a slightly different electronic structure, which is expressed by a lower value of the band gap. However, no significant differences were observed in the spectra recorded by FT-IR, photoluminescence (PL) and electron paramagnetic resonance (EPR) spectroscopic techniques.

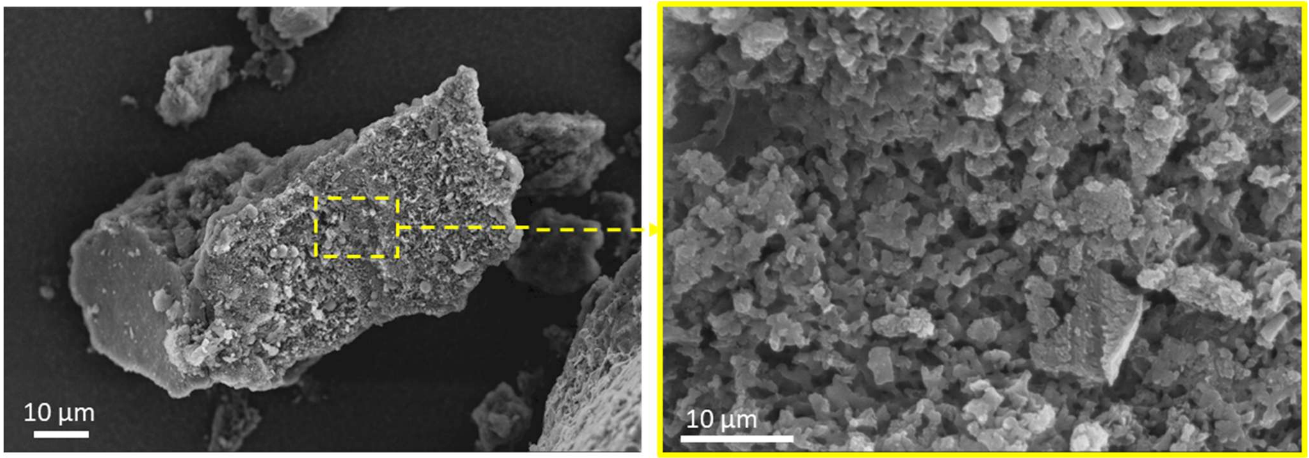

Fig. S12. SEM images of mpg-CN-230 catalyst.

Scanning electron microscopy images (Fig. S12) of mpg-CN-230 also show agglomerated round particles with similar sizes to those observed for mpg-CN-237. No significant differences were observed when comparing the morphology of the two materials.

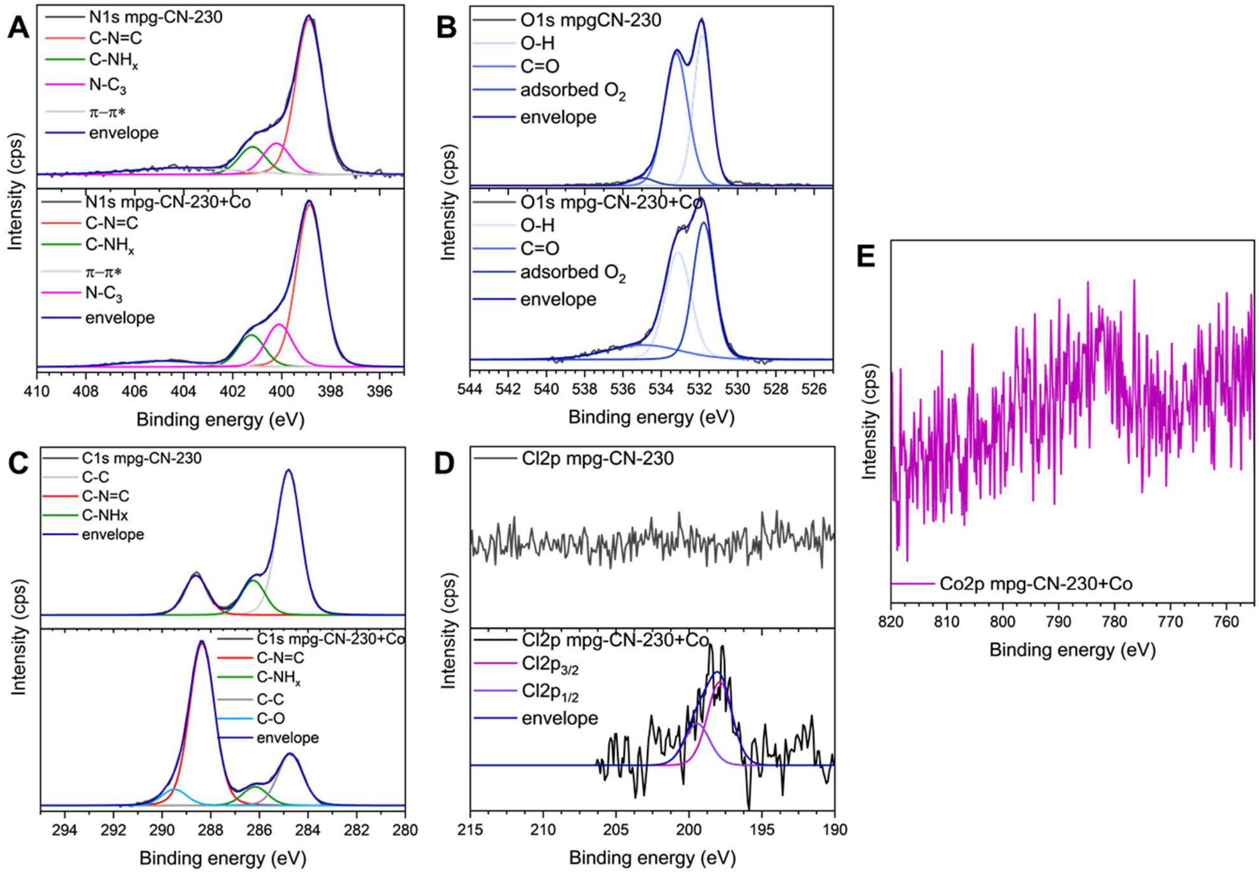

Fig. S13. XPS analysis of mpg-CN-230 and $Co(dmgH)_2PyCl$/mpg-CN-230 N 1s (A), O 1s (B), C 1s (C), Cl 2p (D), Co 2p (E).

The XPS spectra of $Co(dmgH)_2PyCl$/mpg-CN-230 show features similar to those described for $Co(dmgH)_2PyCl$/mpg-CN. Likewise, the introduction of cobalt slightly alters the intensities of the N1s features as well as shifting their position to lower binding energies, suggesting interaction of cobalt with the nitrogen sites. The introduction of cobalt does not drastically change the O1s spectrum, but shifts the positions of the sub-peaks slightly towards lower binding energies, indicating possible interactions with oxygen-containing groups. However, a significant observation is the much larger population for the C-N=C peak in the mpg-CN-230+Co sample compared to the mpg-CN-230 sample. A possible explanation for this is that cobalt may interact preferentially with nitrogen sites, particularly those in the triazine rings (C-N=C), increasing the electron density around these carbon atoms. This interaction could make the C-N=C bonds more prominent in the XPS analysis. The presence of $Co(dmgH)_2PyCl$ can be confirmed by recording the signal typical for Cl 2p, but due to the low signal-to-noise ratio for Co 2p, direct confirmation of the presence of Co was not possible.

Supplementary note 5: Catalytic tests with different mpg-CN material

A systematic screening was performed to optimize the reaction conditions for synthesis using the mpg-CN-230 material. Various parameters were adjusted, including the choice of solvents, concentrations of additives, light sources, and amounts of catalyst.

Table S2. Screening of the reaction conditions using the mpg-CN230 material

Entry	Dev. standard conditions	Yield (%)[a]
1	Standard conditions[b]	**27**
2	DMF, 10 mW cm^{-1}	traces
3	DMSO, 10 mW cm^{-1}	traces
4	Morpholine (0.18 mmol)	27
5	AcOH (10 µl, 0.2 mmol)	24
6	DABCO (100 mg, 0.88 mmol), 16 h	21
7	AcOH (40 µl, 0.7 mmol), 16 h	14
8	DIPEA (26 µL, 0.2 mmol)	4
9	5 mg CN-230	49

[a] GC yield

[b] Reaction conditions: ketone (0.1 mmol, 0.125 mL), morpholine (0.68 mmol, 0.59 mL), mpg-CN 20 mg, $Co(dmgH)_2PyCl$ (0.0012 mmol, 0.5 mg), DABCO (0.22 mmol, 25 mg), glacial AcOH (0.0874 mmol, 0.005 mL), dioxane (1 mL), blue LEDs (λ_{max} = 460 nm, 80 mW cm^{-2}), 25 °C, 24 h, n.d. – not detected.

What stands out in this table is the general pattern of lower aniline yields, even in comparable reaction conditions when mpg-CN-230 is used. That indicates the importance of physicochemical properties of carbon nitride material. From above data it can be concluded that mpg-CN-237's higher proportion of micropores, more amorphous structure with potential defect sites, slight differences in optical properties, and specific functional groups collectively contribute to its enhanced catalytic activity.

Appendix: NMR spectra

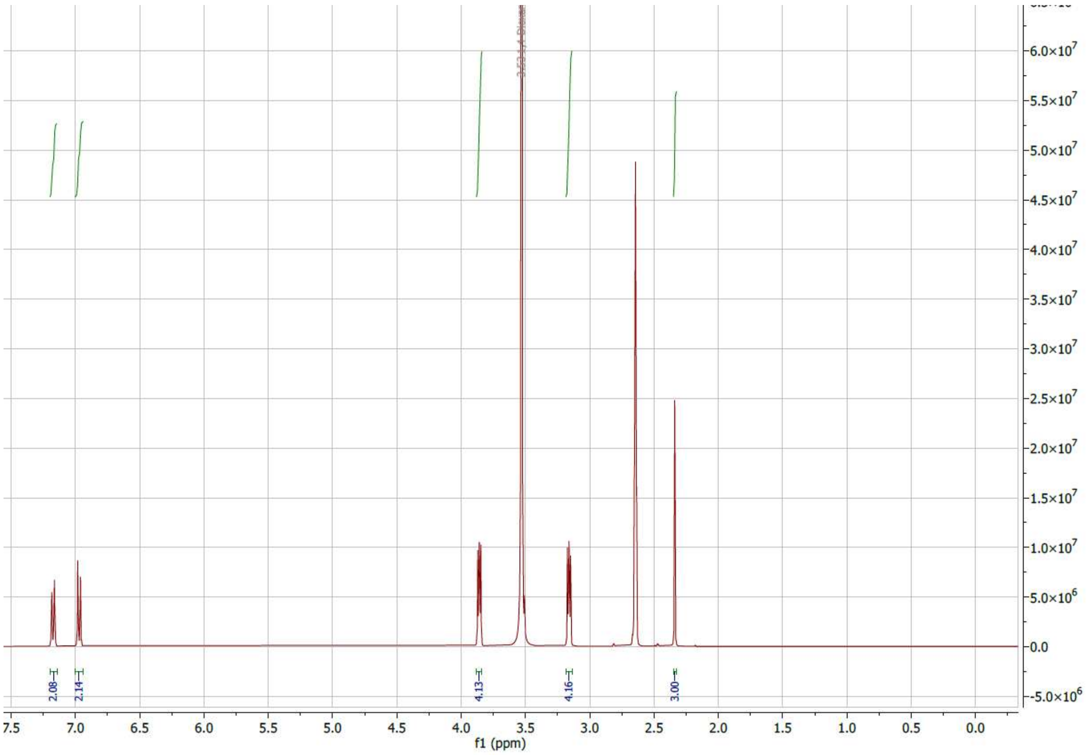

Appendix 1: ^{1}H NMR spectrum of 4-(p-tolyl)morpholine. The compound was synthesized from morpholine and 4-methylcyclohexanone.

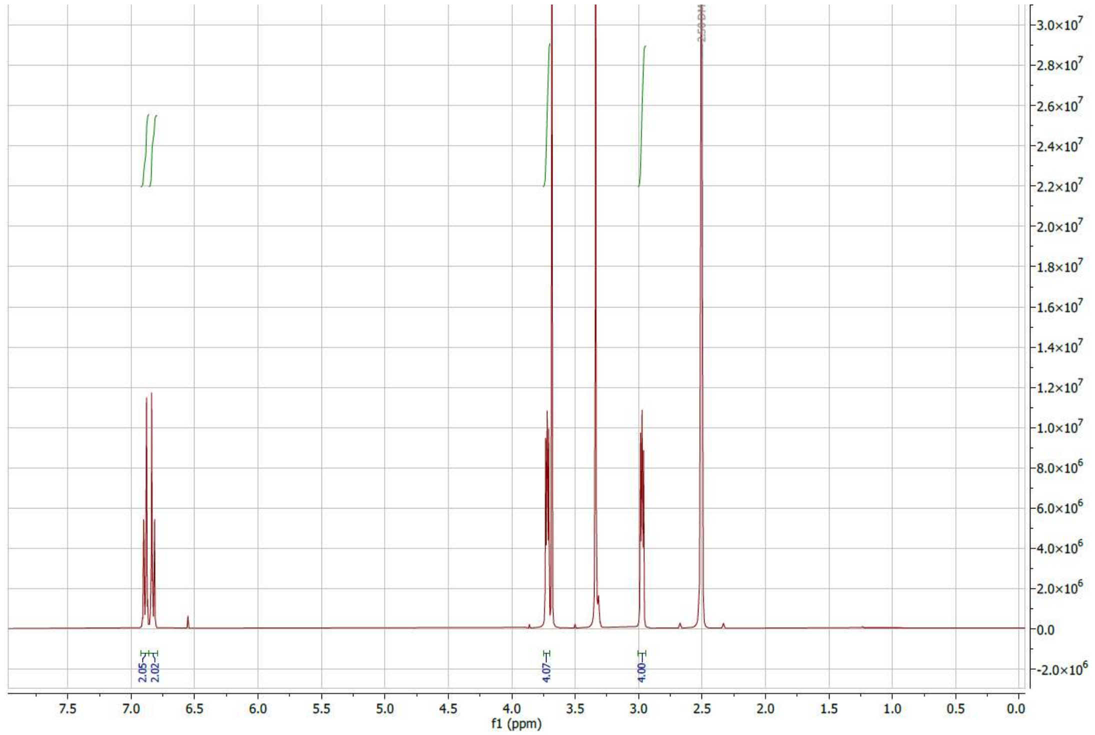

Appendix 2: ^{1}H NMR spectrum of 4-(p-tolyl)morpholine. The compound was synthesized from morpholine and 4-methylcyclohexenone.

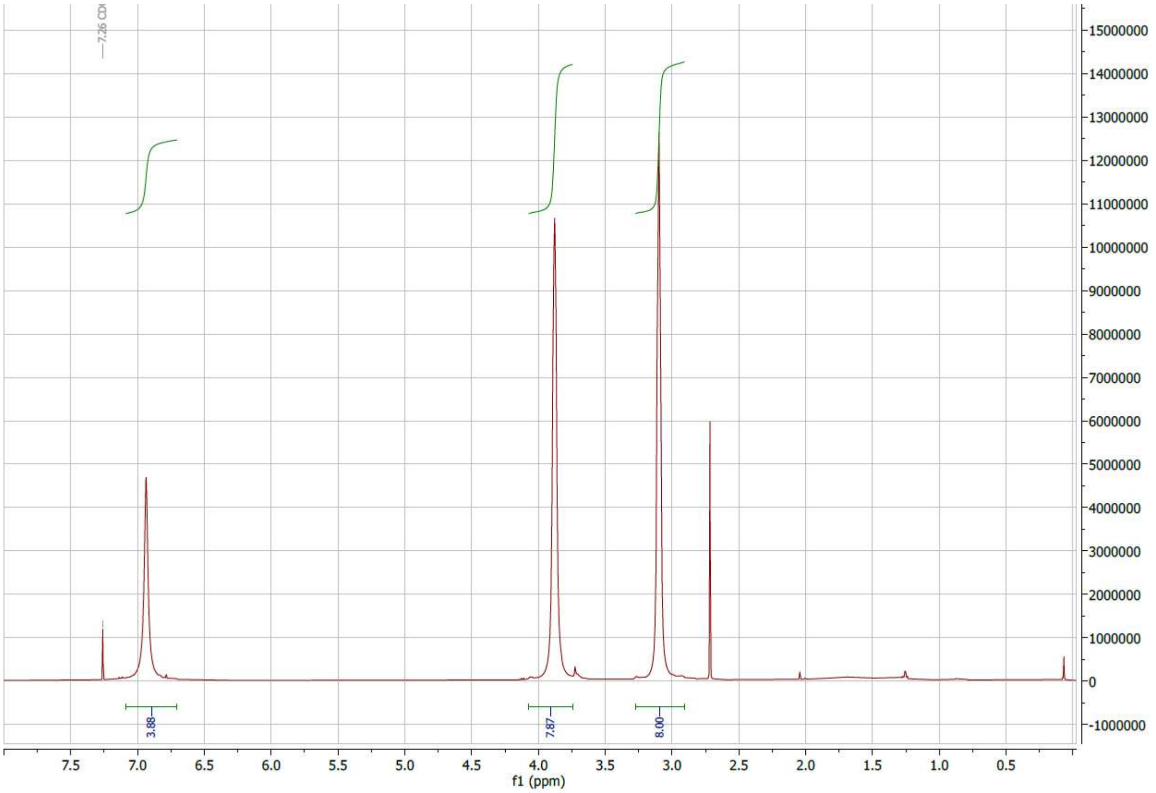

Appendix 3: ^{1}H NMR spectrum of the isolated product of a reaction between morpholine and 1,4-cyclohexanedione.

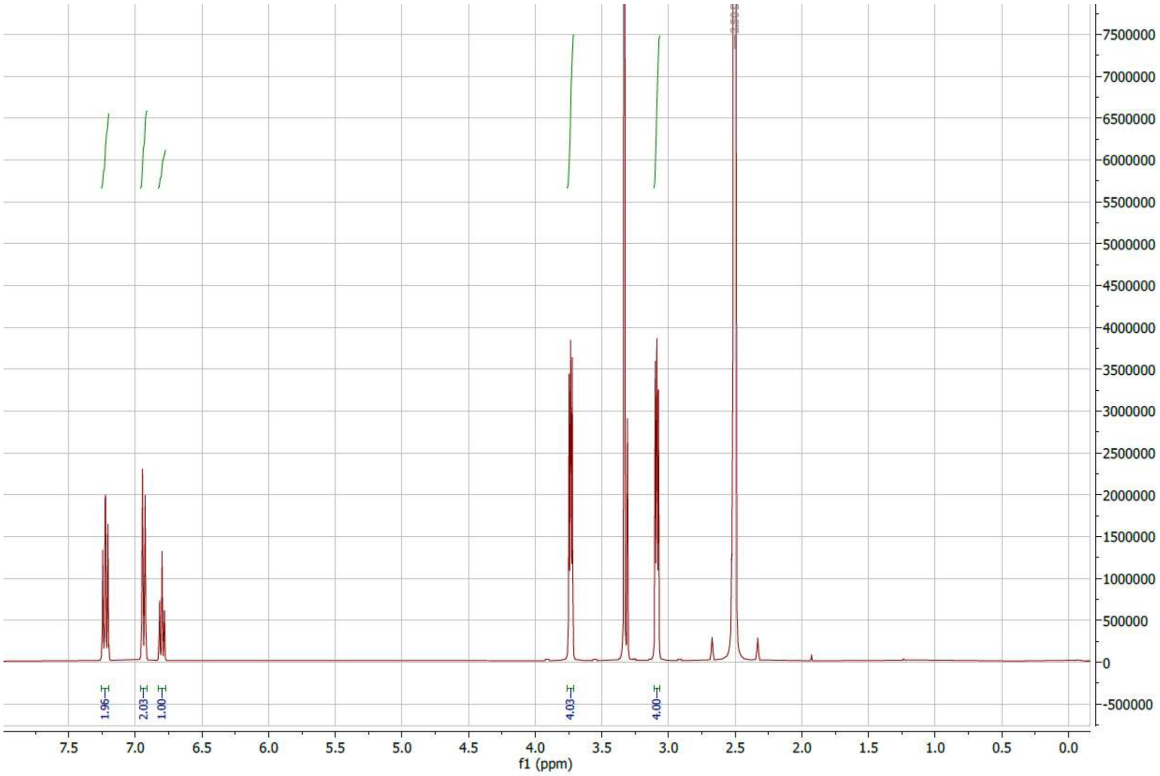

Appendix 4: ^{1}H NMR spectrum of the isolated product of a reaction between morpholine and 2-cyclohexen-1-one

References

(1) Hellgren, N.; Johansson, M. P.; Hjörvarsson, B.; Broitman, E.; Östblom, M.; Liedberg, B.; Hultman, L.; Sundgren, J.-E. Growth, structure, and mechanical properties of CNxHy films deposited by dc magnetron sputtering in N2/Ar/H2 discharges. *Journal of Vacuum Science & Technology A* **2000**, *18* (5), 2349-2358. DOI: 10.1116/1.1286395 (acccessed 2/21/2025).

(2) Soto, G.; Samano, E. C.; Machorro, R.; Farías, M. H.; Cota-Araiza, L. Study of composition and bonding character of CNx films. *Applied Surface Science* **2001**, *183* (3), 246-258. DOI: https://doi.org/10.1016/S0169-4332(01)00567-0.

(3) Wang, X.; Maeda, K.; Thomas, A.; Takanabe, K.; Xin, G.; Carlsson, J. M.; Domen, K.; Antonietti, M. A metal-free polymeric photocatalyst for hydrogen production from water under visible light. *Nature Materials* **2009**, *8* (1), 76-80. DOI: 10.1038/nmat2317.

(4) Cao, S.; Low, J.; Yu, J.; Jaroniec, M. Polymeric Photocatalysts Based on Graphitic Carbon Nitride. *Advanced Materials* **2015**, *27* (13), 2150-2176. DOI: https://doi.org/10.1002/adma.201500033.

(5) Silva, I. F.; Rios, R. D. F.; Savateev, O.; Teixeira, I. F. Carbon Nitride-Based Nanomaterials as a Sustainable Catalyst for Biodiesel Production. *ACS Applied Nano Materials* **2023**, *6* (11), 9718-9727. DOI: 10.1021/acsanm.3c01424.

(6) Silva, I. F.; Teixeira, I. F.; Rios, R. D. F.; do Nascimento, G. M.; Binatti, I.; Victória, H. F. V.; Krambrock, K.; Cury, L. A.; Teixeira, A. P. C.; Stumpf, H. O. Amoxicillin photodegradation under visible light catalyzed by metal-free carbon nitride: An investigation of the influence of the structural defects. *Journal of Hazardous Materials* **2021**, *401*, 123713. DOI: https://doi.org/10.1016/j.jhazmat.2020.123713.

(7) Murugesan, K.; Sagadevan, A.; Peng, L.; Savateev, O.; Rueping, M. Recyclable Mesoporous Graphitic Carbon Nitride Catalysts for the Sustainable Photoredox Catalyzed Synthesis of Carbonyl Compounds. *ACS Catalysis* **2023**, *13* (20), 13414-13422. DOI: 10.1021/acscatal.3c03798.
